# Re-direction of carbon flux to key precursor malonyl-CoA via artificial small RNAs in photosynthetic *Synechocystis* sp. PCC 6803

**DOI:** 10.1186/s13068-018-1032-0

**Published:** 2018-02-05

**Authors:** Tao Sun, Shubin Li, Xinyu Song, Guangsheng Pei, Jinjin Diao, Jinyu Cui, Mengliang Shi, Lei Chen, Weiwen Zhang

**Affiliations:** 1 Laboratory of Synthetic Microbiology, School of Chemical Engineering & Technology, Tianjin University, Tianjin, 300072 People’s Republic of China; 2 Key Laboratory of Systems Bioengineering (Ministry of Education), Tianjin University, Tianjin, 300072 People’s Republic of China; 3 SynBio Research Platform, Collaborative Innovation Center of Chemical Science and Engineering, Tianjin, 300072 People’s Republic of China; 4 School of Environmental Science and Engineering, Tianjin University, Tianjin, 300072 People’s Republic of China; 5 Center for Biosafety Research and Strategy, Tianjin University, Tianjin, People’s Republic of China

**Keywords:** Cyanobacteria, Metabolic regulation, Small RNA tools, Malonyl-CoA

## Abstract

**Background:**

Photosynthetic cyanobacteria have attracted a significant attention as promising chassis to produce renewable fuels and chemicals due to their capability to utilizing solar energy and CO_2_. Notably, the enhancing supply of key precursors like malonyl-CoA would benefit the production of many bio-compounds. Nevertheless, the lacking of genetic tools in cyanobacteria, especially the knockdown strategies for essential pathways, has seriously restricted the attempts to re-direct carbon flux from the central carbohydrate metabolism to the synthesis of bioproducts.

**Results:**

Aiming at developing new genetic tools, two small RNA regulatory tools are reported for the model cyanobacterium *Synechocystis* sp. PCC6803, based on paired termini RNAs as well as the exogenous Hfq chaperone and MicC scaffold (Hfq-MicC) previously developed in *Escherichia coli*. Both regulatory tools functioned well in regulating exogenous reporter gene *lacZ* and endogenous *glgC* gene in *Synechocystis* sp. PCC6803, achieving a downregulation of gene expression up to 90% compared with wildtype. In addition, the Hfq-MicC tool was developed to simultaneously regulate multiple genes related to essential fatty acids biosynthesis, which led to decreased fatty acids content by 11%. Furthermore, aiming to re-direct the carbon flux, the Hfq-MicC tool was utilized to interfere the competing pathway of malonyl-CoA, achieving an increased intracellular malonyl-CoA abundance up to 41% (~ 698.3 pg/mL/OD_730 nm_) compared to the wildtype. Finally, the Hfq-MicC system was further modified into an inducible system based on the theophylline-inducible riboswitch.

**Conclusions:**

In this study, two small RNA regulatory tools for manipulating essential metabolic pathways and re-directing carbon flux are reported for *Synechocystis* sp. PCC6803. The work introduces efficient and valuable metabolic regulatory strategies for photosynthetic cyanobacteria.

**Electronic supplementary material:**

The online version of this article (10.1186/s13068-018-1032-0) contains supplementary material, which is available to authorized users.

## Background

Environmental pollution and climate change resulted from over-consumption of fossil fuels have prompted the researches and development on green fuels and chemicals based on various types of “microbial cell factories” [1, 2]. Among them, photosynthetic cyanobacteria have attracted significant attention as promising chassis for producing green fuels and chemicals due to their capability to utilizing sunlight and CO_2_ as the sole energy and carbon sources, respectively [3]. Up to now, dozens of industrially relevant compounds have been successfully synthesized in several model cyanobacteria, such as *Synechocystis* sp. PCC 6803 (hereafter *Synechocystis*), *Synechococcus elongatus* PCC 7942, and *Synechococcus* sp. PCC 7002 [3].

Nevertheless, compared with other chassis microbes like *Escherichia coli* [4], lacking of genetic tools in cyanobacteria still seriously limits the development and application of cyanobacterial producing systems [5, 6]. In *E. coli*, development and application of various genetic tools such as promoters, riboswitches, ribosome-binding site (RBS) libraries, CRISPR/Cas system (clustered regularly interspaced short palindromic repeats/CRISPR-associated proteins), and small RNA (sRNA) regulatory tools have made it easy to regulate both endogenous and exogenous metabolic pathways to achieve high productivity [7–10]. Among them, genetic tools based on artificial sRNA molecules have showed promising applications [11] as sRNA regulatory tools hardly imposed any metabolic burden on host cells [12]. In addition, the modularity, tunable base-pair complementation, and *trans*-acting ability of sRNA molecules allow for genome-wide regulation of target genes to achieve fine flux control [10, 13, 14]. Moreover, the traditional deletion strategy of the essential genes or pathways is usually lethal to the host cell, while these genes or pathways could be knocked down via sRNA regulatory tools [15].

**Fig. 1:**
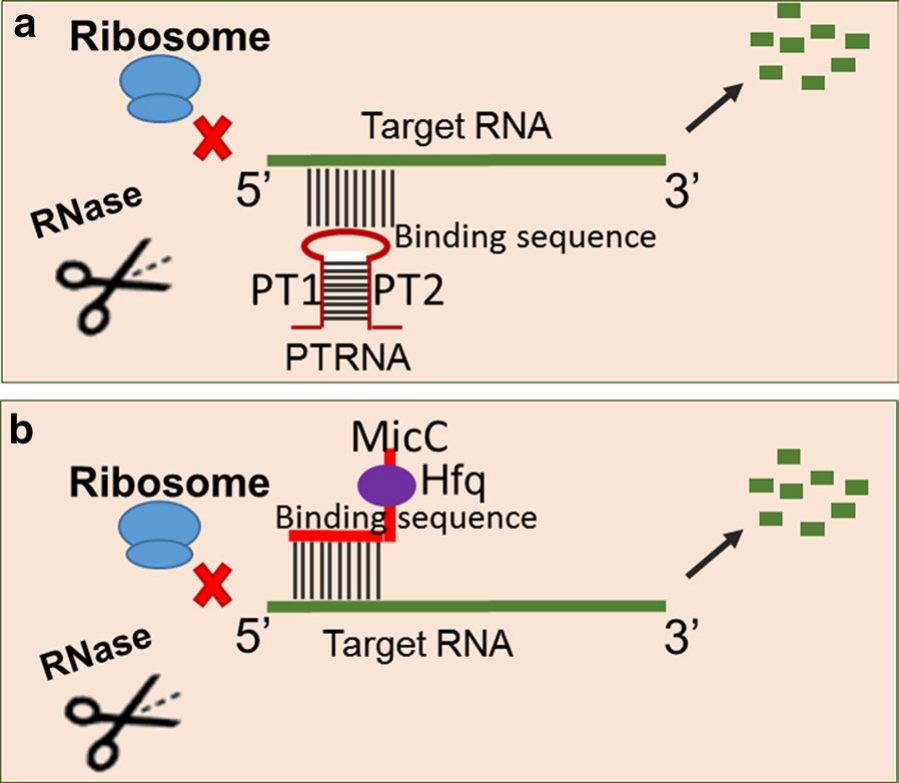
Function and design principles for the PTRNA and Hfq-MicC tools. **a** Schematic of the PTRNA tool. The PTRNA contained a paired terminus composed of PT1 and PT2. The 100 bp binding sequence in PTRNA was designed by choosing the antisense fragment from the translational starting site of the target gene. **b** Schematic of the Hfq-MicC tool. The Hfq-MicC tool contained a 79 bp scaffold named MicC that could be recognized by the chaperone Hfq. The 24 bp binding sequence before MicC was designed by choosing the antisense fragment from the translational starting site of the target gene

In a previous study with *E. coli*, Nakashima et al. [16] reported a sRNA tool based on paired termini antisense RNAs (named as PTRNAs). The tool contained 38 bp flanking inverted repeats to stabilize the middle functioning antisense RNA (asRNA) designed based on the target gene (Fig. 1a), which was able to reach a 78% reduction of the acetate kinase-phosphotransacetylase operon in *E. coli* [16]. In addition, this strategy was later demonstrated effective in regulating various essential genes or pathways [15, 17]. Recently, Yang et al. [18] applied this strategy to regulate fatty acids biosynthesis, successfully enhancing the supply of key intermediate malonyl-CoA and the production of malonyl-CoA-based derivate such as 4-hydroxycoumarin and resveratrol by 4.5-, 2.53-, and 1.70-fold in *E. coli*, respectively. In another study, Na et al. [10] developed a new sRNA regulatory tool based on a chaperone protein Hfq and a well-studied sRNA scaffold from MicC in *E. coli*, in which the designed target-binding region was fused into MicC scaffold and the fused fragment could achieve effective regulation of target genes with the aid of Hfq chaperone (named as Hfq-MicC; Fig. 1b). The chaperone Hfq has three important roles including expediting annealing of sRNA to its target mRNAs, recruiting the major endo-ribonuclease RNase E for cleavage of the targets, and protecting the sRNAs from endonucleolytic cleavage [19–21]. More recently, the applicability of Hfq-MicC tool was also demonstrated in *Clostridium acetobutylicum* [22].

Identification and characterization of cyanobacterial endogenous sRNAs were currently quite limited [23–25], which has restricted the development of sRNA regulatory tools directly using endogenous sRNAs. Up to now, a few studies have focused on the development of sRNAs regulatory tools in cyanobacteria. Recently, post-transcriptional regulatory tool based on *E. coli* IS10 RNA-IN/OUT regulator was introduced into *Synechococcus* sp. PCC 7002 [26], even though inapplicability of this system in controlling endogenous genes makes it challenging for further application in cyanobacteria. To address this issue, in this study, we aim to extend the application of the PTRNA and Hfq-MicC tools in cyanobacteria. First, *lacZ* encoding β-galactosidase and endogenous gene *glgC* related to glycogen biosynthesis were used as the reporter genes to evaluate functionality of PTRNA and Hfq-MicC systems in *Synechocystis*, respectively. In addition, Hfq-MicC tool was utilized to interfere multiple genes in the essential pathways related with fatty acid biosynthesis and malonyl-CoA generation in *Synechocystis*, achieving a re-direction of carbon flux and up to 41% increase (~ 698.3 pg/mL/OD) of intracellular malonyl-CoA abundance compared to wild type (WT). Moreover, the Hfq-MicC tool was modified to be an inducible system based on the theophylline-induced riboswitch. The study introduced efficient and valuable gene regulation strategies for metabolic engineering and synthetic biology in cyanobacteria.

## Results

### Constructing plasmids for gene expression in *Synechocystis*

Aiming at constructing a vector that could replicate in *E. coli* and integrate into the genome of *Synechocystis*, the pTZ57R/T (Thermo Fisher Scientific Inc., CA, USA) was used as a backbone. Two homologous arms based on the neutral integration site of *slr0168* [27], a chloramphenicol-resistant cassette (amplified from pACYC184; hereafter Cm^R^), a strong promoter P*cpc560* [28] or the light-induced P*psb*A2 [29], a multiple cloning site (MCS), and the terminator T*rbcL* [27] were obtained separately, and then successively ligated into backbone of pTZ57R/T, leading to the successful construction of pCP0168 (using P*cpc560*) or pBA0168 (using P*psbA2*), respectively (Additional file 1: Fig. S1A; detailed sequences were summarized in Additional file 2: Table S2). Both constructs were confirmed by Sanger sequencing.

### Constructing plasmids for sRNAs expression in *Synechocystis*

For the construction of sRNA-expressing plasmids, the *slr2030* and *slr2031* genes were chosen as neutral integration sites [30]. Unlike pCP0168, a variant promoter named P*psbAM* without native RBS sequence of P*psbA2* was chosen to prevent the possible translation of sRNAs and ensure the interaction between sRNA and its target mRNA [29]. In addition, a spectinomycin-resistant cassette (hereafter Spe^R^) was utilized (amplified from pXT37b kindly provided by Prof. Xuefeng Lu) [27], leading to pBA3031M (Additional file 1: Fig. S1B).

**Fig. 2:**
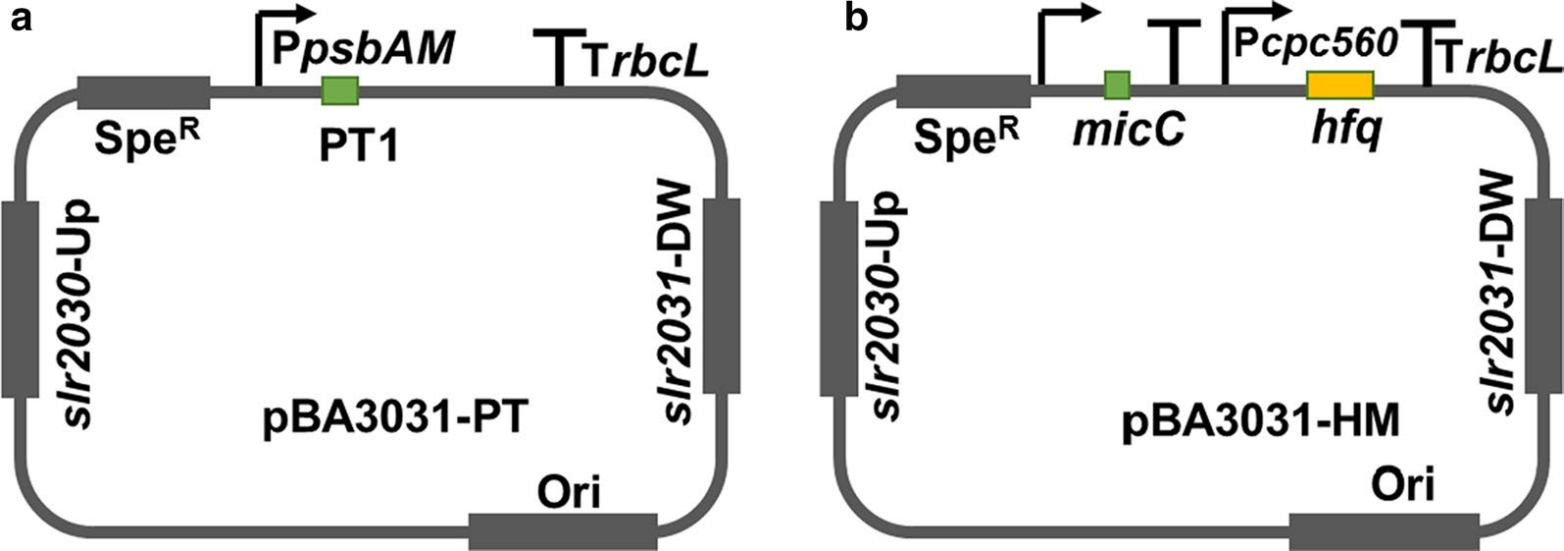
Schematic of the constructed vectors for expressing sRNAs. Detailed sequences were available in Additional file 2: Table S2. **a** Schematic of the pBA3031-PT (Ori was the replicating element ColE1 essential for replication in *E. coli*.). **b** Schematic of the pBA3031-HM. The promoter and terminator for expressing sRNA was the same as the pBA3031-PT

For the construction of PTRNA-expressing plasmid, 38 bp of PT1 sequence (Additional file 2: Table S2) was introduced at a location after P*psbAM* in pBA3031M via inverse PCR. The linear fragment was self-ligated using T4 DNA ligase (Thermo Fisher Scientific Inc., CA, USA), leading to pBA3031-PT (Fig. 2a; Additional file 2: Table S2). The 100 bp binding sequence was obtained by amplifying the antisense fragment from the translational starting site of the target gene [16] (Fig. 1a). The PT2 sequence (Additional file 2: Table S2) was added into the end of the binding sequence via a PCR. The construction was confirmed by Sanger sequencing.

For the construction of Hfq-MicC-expressing plasmid, the P*cpc560*-*hfq*-T*rbcL* cassette (*hfq* was amplified from genome of *E. coli* K12; Additional file 2: Table S2) was achieved by overlapping PCR and then introduced into pBA3031M at a location after T*rbcL*. In addition, the 79 bp fragment of the scaffold from *micC* (synthesized; Additional file 2: Table S2) was introduced into a location after P*psbA2M* by inverse PCR, leading to the pBA3031-HM (Fig. 2b; Additional file 2: Table S2). The 24 bp binding sequence (Fig. 1b) was designed by choosing the antisense fragment from the translational starting site of the target gene and then introduced into pBA3031-HM through inverse PCR and self-ligation [10]. The construction was confirmed by Sanger sequencing.

### Functional validation of PTRNA and Hfq-MicC systems in *Synechocystis* using *lacZ*

**Fig. 3:**
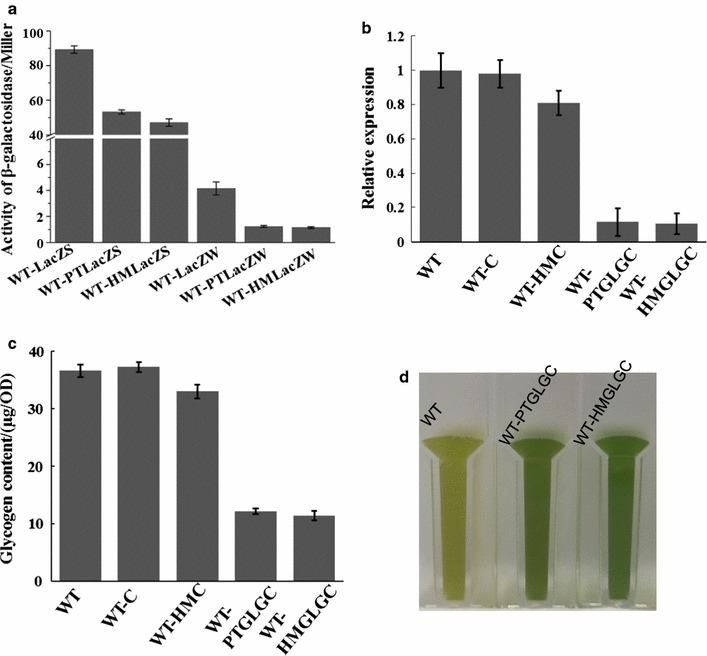
Results of the *β*-Galactosidase activity measurement, qRT-PCR of *glgC*, glycogen quantitation and the nitrogen depletion assay. **a** *β*-Galactosidase activities in WT-LacZS, WT-LacZW, WT-PTLacZS, WT-PTLacZW, WT-HMLacZS, and WT-HMLacZW. The error bar represents the standard deviation of the three biological replicates for each sample. **b** qRT-PCR for the relative mRNA level of *glgC* in WT, WT-C, WT-HMC, WT-PTGLGC, and WT-HMGLGC. The error bar represents the standard deviation of the three technical replicates for each sample. **c** Glycogen quantitation assays in WT, WT-C, WT-HMC, WT-PTGLGC, and WT-HMGLGC. The error bar represents the standard deviation of the three biological replicates for each sample. **d** Nitrogen depletion assay in WT, WT-PTGLGC, and WT-HMGLGC

A widely used reporter gene *lacZ* (Additional file 2: Table S2) encoding *β*-galactosidase was ligated into both pCP0168 and pBA0168, and then introduced into WT, generating WT-LacZS and WT-LacZW, respectively (Table 1). Then, pBA3031-PTLacZ (using PTRNAs targeting *lacZ*) and pBA3031-HMlacZ (using Hfq-MicC targeting *lacZ*) were designed, constructed, and introduced into WT-LacZS and WT-LacZW, leading to WT-PTLacZS, WT-PTLacZW, WT-HMLacZS, and WT-HMLacZW, respectively (Table 1). To evaluate whether PTRNA and Hfq-MicC systems were able to function in *Synechocystis*, activities of *β*-galactosidase were comparatively measured among samples (volume*OD_730 nm_ = 1) of WT, WT-LacZS, WT-LacZW, WT-PTLacZS, WT-PTLacZW, WT-HMLacZS, and WT-HMLacZW. As illustrated in Fig. 3a, *β*-galactosidase activity was much lower in WT-LacZW than that in WT-LacZS due to the weaker strength of P*psbA2* than P*cpc560*. Notably, compared to WT-LacZS, *β*-galactosidase activity was decreased by ~ 40 and ~ 47% in WT-PTLacZS and WT-HMLacZS, respectively; compared to WT-LacZW, *β*-galactosidase activity was, respectively, decreased by ~ 70 and 72% in WT-PTLacZW and WT-HMLacZW (Fig. 3a). The results tentatively demonstrated the functionality of these two sRNA systems in regulating *lacZ* gene expression in *Synechocystis*.

**Table 1:** Strains constructed in this study

Strain	Genotype
WT-LacZS	pCP0168::Pcpc560-*lacZ*-TrbcL; Cm^R^ in WT
WT-LacZW	pCP0168::P*psbA2*-*lacZ*-TrbcL; Cm^R^ in WT
WT-PTLacZS	pBA3031M::P*psbA2* *M*-PT1-*aslacZ1*-PT2-TrbcL; Spe^R^ in WT-LacZS
WT-PTLacZW	pBA3031M::P*psbA2* *M*-PT1-*aslacZ1*-PT2-TrbcL; Spe^R^ in WT-LacZW
WT-HMLacZS	pBA3031M::P*psbA2* *M*-*aslacZ2*-*micC*-TrbcL-Pcpc560-*hfq*-TrbcL; Spe^R^ in WT-LacZS
WT-HMLacZW	pBA3031M::P*psbA2* *M*-*aslacZ2*-*micC*-TrbcL-Pcpc560-*hfq*-TrbcL; Spe^R^ in WT-LacZW
WT-C	pBA3031M:: Spe^R^ in WT
WT-PTGLGC	pBA3031M::PpsbA2 M-PT1-*asglgC1*-PT2-TrbcL; Spe^R^ in WT
WT-HMC	pBA3031M::PpsbA2 M-*asglgC2*-*micC*-TrbcL; Spe^R^ in WT
WT-HMGLGC	pBA3031M::PpsbA2 M-*asglgC2*-*micC*-TrbcL-Pcpc560-*hfq*-TrbcL; Spe^R^ in WT
WT-HMFA	pBA3031M::PpsbA2 M-*asslr1511*-*micC*-TrbcL-PpsbA2 M-*asslr1332*-*micC*-TrbcL-PpsbA2 M-*assll1069*-*micC*-TrbcL-Pcpc560-*hfq*-TrbcL; Spe^R^ in WT
WT-HMMA1	pBA3031M::PpsbA2 M-*asslr2023*-*micC*-TrbcL-PpsbA2 M-*asslr1511*-Pcpc560-*hfq*-TrbcL; Spe^R^ in WT
WT-HMMA2	pBA3031M::PpsbA2 M-*asslr2023*-*micC*-TrbcL-PpsbA2 M-*asslr1511*-*micC*-TrbcL-PpsbA2 M-*asslr1332*-*micC*-TrbcL-PpsbA2 M-*assll1069*-*micC*-TrbcL-Pcpc560-*hfq*-TrbcL; Spe^R^ in WT
WT-HMLacZS2	pTR3031M::PpsbA2 M-*aslacZ2*-*micC*-TrbcL-Ptrc-riboswitch-*hfq*-TrbcL; Spe^R^ in WT-LacZS
WT-HMMA1(the*)	pBA3031M::PpsbA2 M-*asslr2023*-*micC*-TrbcL-PpsbA2 M-*asslr1511*-Ptrc-riboswitch-*hfq*-TrbcL; Spe^R^ in WT

### Functional validation of PTRNA and Hfq-MicC tools in *Synechocystis* using *glgC*

Furthermore, we chose the native *glgC* gene of *Synechocystis* involving glycogen biosynthesis to evaluate the functional performance of PTRNA and Hfq-MicC tools on regulating the endogenous gene. The plasmid pBA3031-PTGLGC and pBA3031-HMGLGC with the binding sequence targeting *glgC* were designed and constructed based on PTRNA and Hfq-MicC tool, respectively, resulting in WT-PTGLGC and WT-HMGLGC, respectively (Table 1). Meanwhile, two strains, i.e., WT-C and WT-HMC, were constructed as controls (Table 1). WT-C obtained an empty vector of pBA3031M; thus, only the neutral site was replaced by Spe^R^ compared to WT. In addition, WT-HMC only lacked the P*cpc560*-*hfq*-T*rbcL* cassette compared with WT-HMGLGC. The expression of sRNAs in WT-HMC, WT-PTGLGC and WT-HMGLGC was validated using RT-PCR (Additional file 1: Fig. S2). Consistent with the previous studies [16, 31], the expression level of sRNAs was more abundant in WT-PTGLGC and WT-HMGLGC than that in WT-HMC due to the protection by the paired termini or Hfq (Additional file 1: Fig. S2A). To investigate the performance of PTRNA and Hfq-MicC systems in regulating *glgC* gene expression, qRT-PCR assay (quantitative real-time polymerase chain reaction) was utilized for WT, WT-C, WT-HMC, WT-PTGLGC, and WT-HMGLGC. As illustrated in Fig. 3b, the mRNA level of *glgC* in WT-PTGLGC was decreased by about 89% compared to that in WT, suggesting a very high efficiency of the PTRNA tool. In addition, without the chaperone Hfq, the mRNA level of *glgC* in WT-HMC could also achieve a 20% decrease compared to that in WT (Fig. 3b), possibly due to the *trans*-regulatory role of sRNA alone. Notably, with the aid of Hfq, a 90% downregulation of *glgC* gene expression was observed in WT-HMGLGC compared to that in WT (Fig. 3b), indicating the functionality of Hfq-MicC tool in *Synechocystis* and the essential role of Hfq. Then, the glycogen quantitation assays were performed in the five strains including WT, WT-C, WT-HMC, WT-PTGLGC, and WT-HMGLGC. As shown in Fig. 3c, though no significant difference in glycogen content was detected in WT-C and WT-HMC compared to WT, the glycogen content in WT-PTGLGC and WT-HMGLGC was significantly decreased by 66.7 and 68.8% compared to WT at 72 h. Finally, as it is well known that the *glgC*-deficient strain had an obvious phenotype resistant to the bleaching process under nitrogen starvation [30, 32], nitrogen depletion assay was performed to verify the decrease of glycogen in WT-PTGLGC and WT-HMGLGC. As expected, both WT-PTGLGC and WT-HMGLGC showed a non-bleaching phenotype compared to WT (Fig. 3d). Though the non-bleaching phenotype was not completely inhibited in WT-PTGLGC and WT-HMGLGC, it could last for at least 2 days or more than 2 weeks under shaking or static culture condition, respectively. Altogether, based on both studies on *lacZ* and *glgC*, the results showed that the Hfq-MicC tool was able to function better than the PTRNA tool in *Synechocystis*, as a higher percentage of downregulation of target gene was observed. Given its efficiency, the Hfq-MicC tool was chosen for the following application and optimization. In addition, as WT-C showed no difference from WT in the above assays, WT alone was thus used as the control for the following experiments.

### Knockdown of essential fatty acid biosynthesis pathway using Hfq-MicC tool

**Fig. 4:**
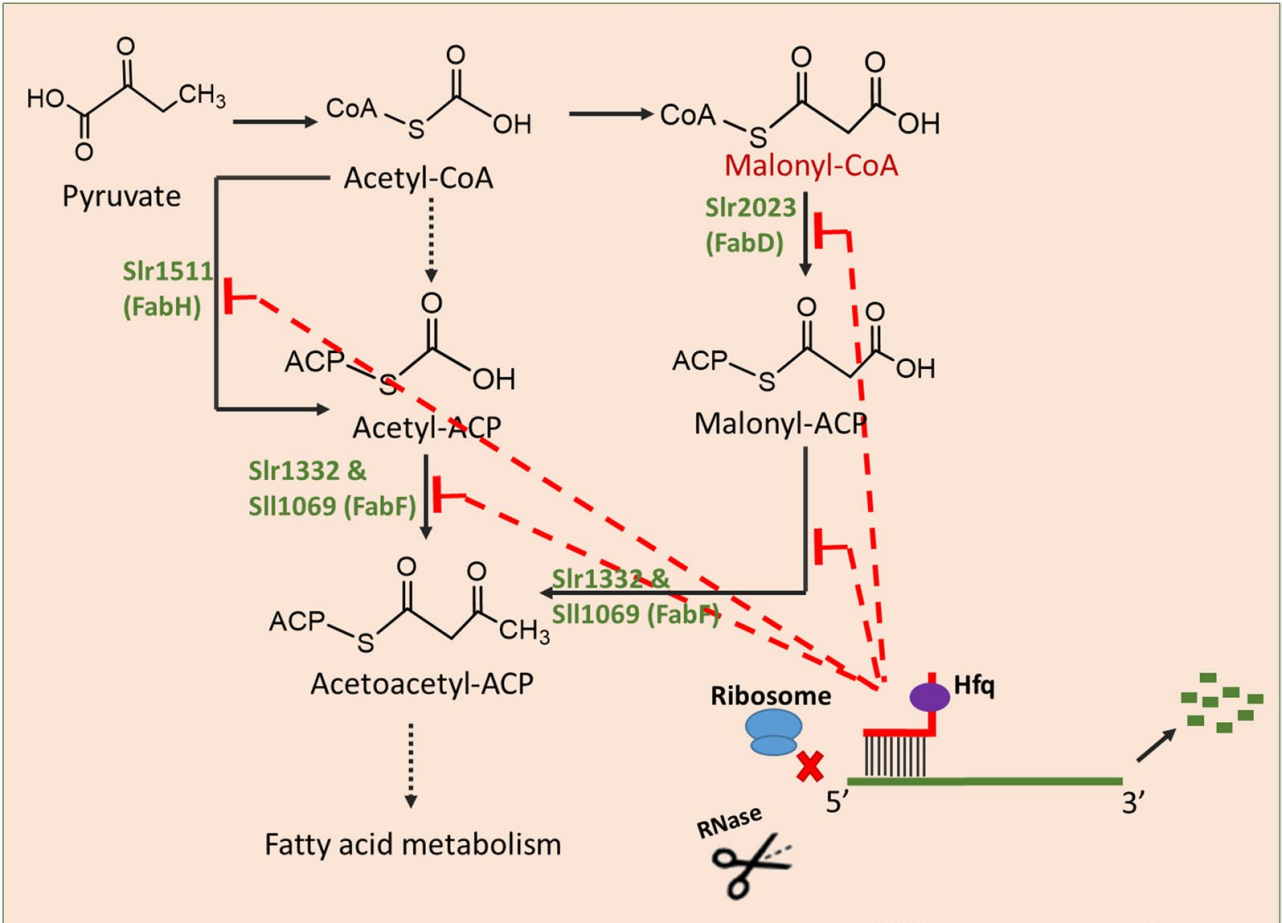
Fatty acid biosynthesis, malonyl-CoA generation as well as the metabolic regulation strategy in *Synechocystis*. Fatty acid biosynthesis depends on the key precursor including acetyl-CoA and malonyl-CoA as well as the key enzymes like FabH, FabF, and FabD. Downregulation of the expression of the key genes using the Hfq-MicC tool would alter the generation of fatty acid and promote the accumulation of malonyl-CoA

**Fig. 5:**
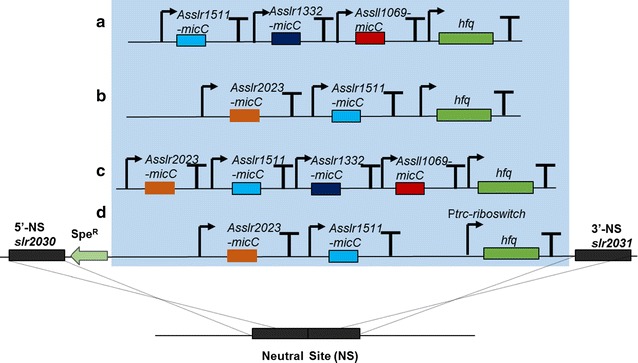
Constructed strains using the Hfq-MicC tool. P*psbA2* *M* promoter was used for expressing all sRNAs, while P*cpc560* was utilized to express the chaperone *hfq* except WT-HMMA1(the*). T*rbcL* was used as the terminators. **a** Schematic of the WT-HMFA strain, in which *slr1511*, *slr1322,* and *sll1069* were downregulated. **b** Schematic of the WT-HMMA1 strain, in which *slr2023* and *slr1511* were downregulated. **c** Schematic of the WT-HMMA2 strain, in which *slr2023*, *slr1511*, *slr1322,* and *sll1069* were downregulated. **d** Schematic of the WT-HMMA1(the*) strain, in which *slr2023* and *slr1511* were downregulated. A modified promoter P*trc* contained a theophylline-responsive riboswitch was used to control the expression of *hfq*

**Fig. 6:**
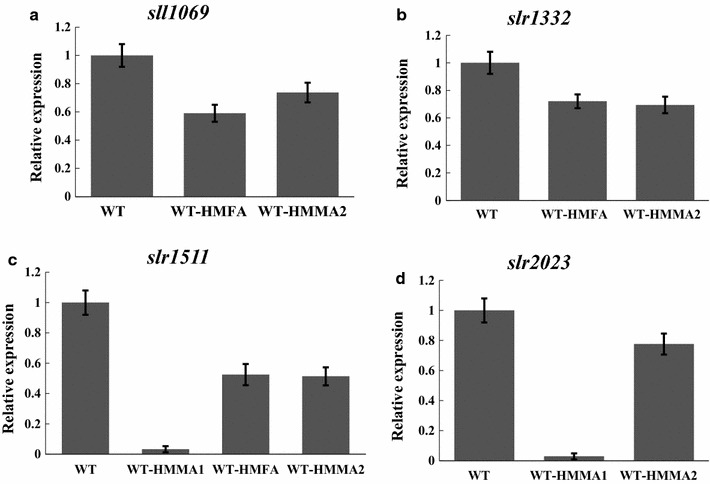
qRT-PCR assays for detecting the mRNA levels. The error bar represents the standard deviation of the three technical replicates for each sample. **a** Relatively transcriptional level of *sll1069* in WT, WT-HMFA, and WT-HMMA2. **b** Relatively transcriptional level of *slr1332* in WT, WT-HMFA, and WT-HMMA2. **c** Relatively transcriptional level of *slr1511* in WT, WT-HMFA, WT-HMMA1, and WT-HMMA2. **d** Relatively transcriptional level of *slr2023* in WT, WT-HMMA1, and WT-HMMA2

**Fig. 7:**
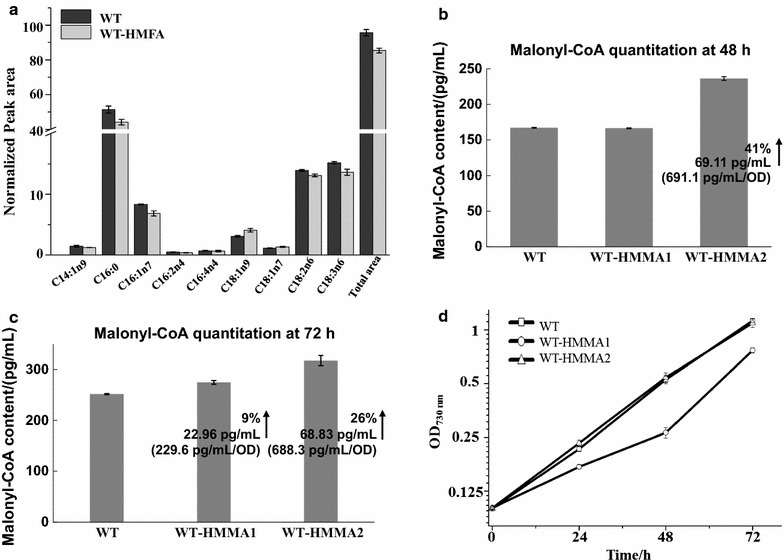
Results of the lipid profiles, malonyl-CoA quantitation, and the growth patterns. **a** The lipid profiles for identified fatty acids in WT and WT-HMFA. The error bar represents the standard deviation of the three biological replicates for each sample. (Take “*C*(*a*):(*b*)*n*(*c*)” as an example, “*a*” means the total numbers of “*C*” and *b* means the number of existing “*C* = *C*” while “c” means the location of the first “*C* = *C*”.) **b** Malonyl-CoA quantitation in WT, WT-HMMA1 and WT-HMMA2 at 48 h. The error bar represents the standard deviation of the three biological replicates for each sample. **c** Malonyl-CoA quantitation in WT, WT-HMMA1 and WT-HMMA2 at 72 h. The error bar represents the standard deviation of the three biological replicates for each sample. **d** Growth patterns of WT, WT-HMMA1, and WT-HMMA2. The error bar represents the standard deviation of the three biological replicates for each sample

Utilizing the Hfq-MicC tool established, we aimed at simultaneously regulating multiple genes of *Synechocystis*. The fatty acid biosynthetic pathway was selected, as it is essential and cannot be knocked out using the traditional manipulation strategy [18]. FabH encoding gene *slr1511* related to initiation, and FabF encoding genes *sll1069* and *slr1332* related to elongation of fatty acid biosynthesis process were selected as target genes [33]. The WT-HMFA strain was constructed (Table 1; Fig. 5a), targeting *sll1069*, *slr1332,* and *slr1511* genes involved in fatty acid biosynthesis (Fig. 4). The expression of sRNAs in WT-HMFA was validated using RT-PCR (reverse transcription polymerase chain reaction) (Additional file 1: Fig. S2). First, the qRT-PCR assay was carried out to determine the mRNA abundances of three target genes in WT-HMFA. As illustrated in Fig. 6a–c, transcriptional level of *sll1069*, *slr1332,* and *slr1511* was, respectively, decreased by 41, 28, and 48% compared to that in WT. In addition, the lipid profiles between WT and WT-HMFA were comparatively measured using a GC analysis, and the results showed that most of the detected fatty acids showed a decreased peak area in WT-HMFA compared to that in WT, leading to approximately 11% decrease in total fatty acid content (Fig. 7a). Notably, C16:0, C16:1n7, C16:2n4, and C18:3n6 were significantly decreased by 14, 18, 25, and 10% in WT-HMFA, respectively, compared with that in WT. Together, these results demonstrated functionality of the Hfq-MicC tool in simultaneously regulating multiple genes of the fatty acid biosynthetic pathway in *Synechocystis*.

### Re-direction of carbon flux from fatty acid biosynthesis to key precursor malonyl-CoA

Malonyl-CoA is an important precursor for many bioproducts like polyketides, flavonoids, 3-hydroxypropionic acid (3-HP), 4-hydroxycoumarin, tetracyclines, and doxorubicin [18, 34], and thus, the enhancing cellular pool of malonyl-CoA could potentially benefit the production for malonyl-CoA-derived products. The previous studies have suggested fatty acid biosynthesis as the major competitive pathway for malonyl-CoA generation [18]. To re-direct more carbon sources towards malonyl-CoA, we designed two other strains based on WT-HMFA. First, two genes, *slr2023* (encoding FabD) and *slr1511* (encoding FabH), were regulated by the Hfq-MicC tool in WT-HMMA1 as FabD and FabH were the key enzymes to catalyze the conversion of malonyl-CoA and its precursor acetyl-CoA into fatty acid synthesis, respectively (Table 1; Figs. 4 and 5b). Second, *sll1069* and *slr1332* putatively encoding FabF were also targeted with the Hfq-MicC tool along with *slr1511* and *slr2023* in WT-HMMA2 to decrease the consumption of both malonyl-CoA and its precursor acetyl-CoA (Table 1; Fig. 4 and 5c). The expression of sRNAs in WT-HMMA1 and WT-HMMA2 was validated using RT-PCR (Additional file 1: Fig. S2). Similarly, the qRT-PCR was performed first to validate the knockdown effect of the Hfq-MicC tool on all four target genes. Notably, as much as 96 and 97% mRNA of *slr1511* and *slr2023* were, respectively, downregulated in WT-HMMA1 (Fig. 6c, d), while 26, 49, 22, and 31% decrease were measured for *sll1069*, *slr1332, slr1511,* and *slr2023* in WT-HMMA2, respectively (Fig. 6). In addition, to determine the intracellular malonyl-CoA abundance, a bacterial malonyl-CoA quantitation kit was used for WT, WT-HMMA1, and WT-HMMA2. Samples (volume*OD_730 nm_ = 0.5) for analysis were collected at middle-exponential phase (48 h) and end-exponential phase (72 h). The results showed a significant increase of malonyl-CoA content up to 69.11 and 69.83 pg/mL under all tested conditions in WT-HMMA2 for both 48 and 72 h, indicating an enhancing supply of malonyl-CoA by 41% (~ 691.1 pg/mL/OD_730 nm_) and 26% (~ 698.3 pg/mL/OD_730 nm_), respectively (Fig. 7b, c). Interestingly, although gene expression of *slr1511* and *slr2023* was almost fully blocked in WT-HMMA1, the malonyl-CoA content in WT-HMMA1 was only slightly increased by 9% (i.e., 22.96 pg/mL; ~ 229.6 pg/mL/OD_730 nm_) at 72 h when compared with WT (Fig. 7c). Nevertheless, the results clearly demonstrated the application of the Hfq-MicC tool in metabolic regulation for *Synechocystis*.

### Development of an inducible Hfq-MicC system

Although regulation mediated by the sRNA tools has not totally blocked the expression of essential genes or pathways, the over downregulation could also affect cell growth and metabolism due to the essential roles played by the targets in supporting cell survival. Previously, growth inhibition was also observed in *E. coli* when PTRNA was used to achieve the knockdown of *fab*I related to fatty acid metabolism [16]. In this study, as *slr1511* and *slr2023* were almost fully blocked (up to 96 and 97%, respectively) in HMMA-1, we also observed severe growth inhibition (Fig. 7d) and bleaching phenotype (Additional file 1: Fig. S5) for the strain, such “negative effects” could restrict our goal of improving malonyl-CoA accumulation in cyanobacterial chassis for its biotechnological application.

**Fig. 8:**
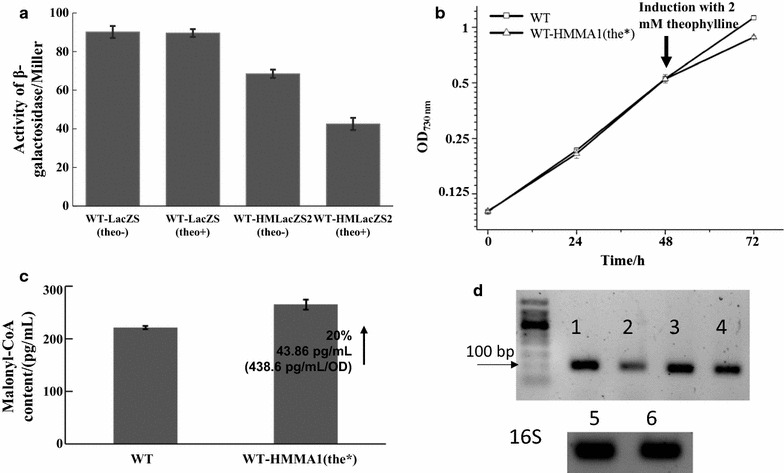
Results of the *β*-galactosidase activity measurement, growth patterns, malonyl-CoA quantitation, RT-PCR. **a** *β*-Galactosidase activities in WT-LacZS and WT-HMLacZS without (shown as “theo−”) or with (shown as “theo+”) 2 mM theophylline induction. The error bar represents the standard deviation of the three biological replicates for each sample. **b** Growth patterns for WT and WT-HMMA1(the*). Both WT and WT-HMMA1(the*) were induced with 2 mM theophylline at 48 h. The error bar represents the standard deviation of the three biological replicates for each sample. **c** Malonyl-CoA contents of WT and WT-HMMA1(the*) at 72 h. The error bar represents the standard deviation of the three biological replicates for each sample. **d** RT-PCR for WT-HMMA1(the*) with or without induction. Lane 1–6: 1 and 3 were the expression level of *Asslr1511*-*micC* and *Asslr2023*-*micC* with 2 mM theophylline induction, respectively; 2 and 4 were the expression level of *Asslr1511*-*micC* and *Asslr2023*-*micC* without induction, respectively; 4 and 6 were the expression level of 16S rRNA in WT-HMMA1(the*) with or without induction, respectively

To address the issue, efforts can be made to make the Hfq-MicC system more controllable, so that normal biomass accumulation without interference is allowed at the early stage of cultivation, while malonyl-CoA accumulation is induced for biosynthesis of chemicals at the late stage of cultivation or anytime. Towards this goal, first, the promoter of P*cpc560*-*hfq*-TrbcL cassette in pBA3031-HM was replaced with the P*trc* containing a theophylline-induced riboswitch [35] (the new vector was named as pBA3031-HM(the*); Additional file 1: Fig. S6, Additional file 2: Table S2). Then, the binding sequence for *lacZ* was ligated into pBA3031-HM(the*) and introduced into WT-LacZS, leading to WT-HMLacZS2 (Table 1). The *β*-galactosidase activity was comparatively measured among WT, WT-LacZS, and WT-HMLacZS2. As shown in Fig. 8a, *β*-galactosidase activity was decreased by ~ 25% in WT-HMLacZS2 without induction. As a previous study has showed that the theophylline-induced riboswitch can tightly control protein expression in *Synechocystis* [36], the un-induced expression of Hfq can be neglected. Therefore, it was expected that the leakage in our system might be mostly due to the regulatory role of sRNA, as its binding sequence with 24 nucleotides complementary to the target mRNA could act as an antisense RNA [22]. After induction with 2 mM theophylline (no toxicity of theophylline on the growth of WT at this concentration as shown in Additional file 1: Fig. S7) [36], the *β*-galactosidase activity was decreased by ~ 52%, suggesting the good performance of theophylline-induced riboswitch in controlling the translation of *hfq*. Based on the results using *lacZ*, a modified strain of WT-HMMA1 named WT-HMMA1(the*) was constructed, in which the expression of *hfq* was controlled by the theophylline-induced riboswitch (Table 1; Fig. 5d). As expected, the growth inhibition observed previously was disappeared without induction before 48 h (Fig. 8b). After induction with 2 mM theophylline, the slower growth of WT-HMMA1(the*) than WT was observed at 72 h (Fig. 8b), which was in accordance with the inhibited growth of WT-HMMA1. Notably, an obvious 20% increase of 43.86 pg/mL of malonyl-CoA content was obtained for WT-HMMA1(the*) compared to WT at 72 h (Fig. 8c) (~ 438.6 pg/mL/OD_730 nm_), which was much higher that found in the WT-HMMA1 containing a non-inducible system (i.e., 9%). The results demonstrated the functionality of the inducible Hfq-MicC system in regulating essential pathways in *Synechocystis*.

**Fig. 9:**
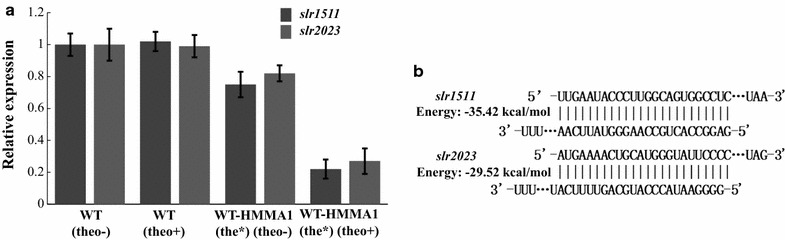
Results of the qRT-PCR and the binding energy calculation. **a** Results of qRT-PCR for the relative mRNA levels of *slr1511* and *slr2023* in WT and WT-HMMA1(the*) without (shown as “theo−”) or with (shown as “theo+”) 2 mM theophylline induction, respectively. The error bar represents the standard deviation of the three technical replicates for each sample. **b** Binding energy between the sRNA and the corresponding target for *slr1511* and *slr2023*. The binding energy was calculated by the IntaRNA using the unique sequences of sRNA and its target mRNA

To evaluate the induction of Hfq, regulation efficiency as well as the potential leakage in WT-HMMA1(the*), sodium dodecyl sulfate polyacrylamide gel electrophoresis (SDS-PAGE), RT-PCR, and qRT-PCR assays were performed among WT and WT-HMMA1(the*) without or with induction. First, consistent with the previous report [36], the expression of Hfq was not detected in WT-HMMA1(the*) without induction, while a significant signal could be obtained after 2 mM theophylline induction (Additional file 1: Fig. S8). In addition, the expression level of *Asslr1511*-*micC* and *Asslr2023*-*micC* in WT-HMMA1(the*) without induction was significantly lower than that in WT-HMMA1(the*) after induction due to the losing protection from Hfq (Fig. 8d). Moreover, as shown in Fig. 9a, nearly 80% mRNA of both *slr1511* and *slr2023* was knocked down after induction. However, a 28% and 19% decrease were also observed for *slr1511* and *slr2023* without induction, respectively, suggesting the possible leakage of the current system. As mentioned above, we suspected that the leakage was mainly from the role of sRNA without *hfq*. Thus, the lower of the binding energy between sRNA and its target, the higher of the leakage should be investigated. To evaluate this hypothesis, the IntaRNA tool developed for predicting interaction between sRNA and target gene for cyanobacteria was utilized [37, 38]. Consistently, the results showed that the binding energy was − 35.42 kcal/mol for *slr1511* and its related sRNA, which was lower than that for *slr2023* and its related sRNA (Fig. 9b; related sequences were listed in Additional file 2: Table S3). To make sure that the leakage problem was controllable, we further calculated the binding energy for all 3178 genes of the *Synechocystis* genome using their mRNA sequences and unique binding sRNAs for each gene. Interestingly, binding energy of most genes (~ 82%) was above − 35.42 kcal/mol (Additional file 1: Fig. S9), while only ~ 3% genes were found with a binding energy less than − 40 kcal/mol (Additional file 1: Fig. S9). The analysis results implied that the leakage could be controlled below 30% for most of the target genes in the *Synechocystis* genome using pBA3031-HM(the*) under our tested conditions.

## Discussion

In recent years, various genetic tools have gradually become available in cyanobacteria [26, 28, 30, 35, 39]. Nevertheless, inherent defects of the current genetic tools sometimes made it not suitable for metabolic engineering and synthetic biology. The expression levels of non-inherent heterologous genes cloned in plasmid vectors can be tuned quite efficiently using these traditional tools and methods like promoters, RBS sequences, and riboswitches, but those of inherent chromosomal genes are still laborious and time-consuming to manipulate [22]. In addition, transcriptional blockage of essential genes or pathways mediated by CRISPR/Cas system could be fatal to cyanobacteria [40]. In this study, aiming to extend the cyanobacterial genetic tool libraries, we developed two sRNA regulatory tools for the model cyanobacterium *Synechocystis*, based on the PTRNA and Hfq-MicC tools of *E. coli*, respectively. The results demonstrated their utilization in regulating single or multiple gene(s) in *Synechocystis*. Compared to other genetic tools available in cyanobacteria, the PTRNA and the Hfq-MicC tool are suitable for regulating both endogenous and exogenous gene(s), especially metabolically essential gene(s).

Primary role of fatty acids is to act as the hydrophobic component of the membrane lipids in both eukaryotes and prokaryotes [33]. Fatty acid synthases are responsible for the biosynthesis of fatty acids, extending its length by two carbon units every cycle [41]. Meanwhile, some of the intermediate metabolites are important precursors for synthesis of many bioproducts [3]. However, as fatty acid biosynthesis could compete carbon flux with synthesis of these bioproducts, it is necessary to develop technologies to regulate the essential fatty acid pathways, so that more carbon flux can be directed to bioproducts. Previously, Yang et al. [18] utilized PTRNAs to regulate the key genes *fabD*, *fabB*, *fabF,* and *fabH* involving fatty acids biosynthesis in *E. coli,* successfully decreasing the fatty acid generation, and enhancing the supply of malonyl-CoA up to fivefold. However, due to the lack of suitable genetic tools for knocking down essential genes, regulation of fatty acid biosynthesis can hardly be performed in cyanobacteria. In cyanobacteria, FabH was claimed as the sole rate-limiting enzyme, and the ketosynthase FabF was found to be essential for fatty acid synthase turnover [42, 43]. In accordance to these early results, interference of FabH (encoded by *slr1511*) and FabF (encoded by *sll1069* and *slr1332*) in WT-HMFA by sRNA tools developed in this study led to decreased contents of fatty acids than WT. In addition, as demonstrated by the qRT-PCR analysis, the Hfq-MicC tool was efficient to interfere either one (WT-HMGLGC, Fig. 3b) or two targets (WT-HMMA1, Fig. 6c, d), with greater than 90% of gene expression decreased. It worth noting that with the number of target genes increased to three (in WT-HMFA) or four (in WT-HMMA2), the interference efficiency of the Hfq-MicC tool was declining (Fig. 6). As the chaperone Hfq is essential for the Hfq-MicC system, one possible explanation for the declined efficiency with more target genes (i.e., > 3) could be due to the insufficient supply of Hfq chaperone, which was in consistent with the overexpression of synthetic sRNAs resulting in reduction of available Hfq proteins in *E. coli* [44]. In addition, another possible explanation could be due to the different expression levels of sRNAs in different constructed strains, although no significant differences could be seen from the RT-PCR assays (Additional file 1: Fig. S2B). Nevertheless, to validate these assumptions, in the future, the expression level of Hfq or sRNAs should be enhanced using stronger promoters or duplicate copies when regulating multiple genes to compare with the current one.

Malonyl-CoA served as a major building block for a variety of relevant derivatives like polyketides, flavonoids, and fatty acid-derived products [45]. As the generation of malonyl-CoA from acetyl-CoA was catalyzed by acetyl-CoA carboxylase (ACC) [45], manipulation of ACC has previously been demonstrated to be an effective strategy to enhance the malonyl-CoA pool in cells [46]. In our previous study, overexpression of four key genes encoding AccA, AccB, AccC, and AccD was also able to improve the malonyl-CoA supply, eventually leading to an increased 3-HP production up to 4.5-fold [34]. In addition, by engineering phospholipid synthesis transcriptional regulators in *S. cerevisiae*, a significant increase of malonyl-CoA content in the engineered strains and ninefold increase of the titer of malonyl-CoA-derived 3-HP were achieved [47]. In this work, in accordance with the previous study using PTRNA tool to regulate the malonyl-CoA supply [18], we decreased the flux from both acetyl-CoA and malonyl-CoA to fatty acids biosynthesis using the Hfq-MicC tool to downregulating two (*slr1511* and *slr2023*) or four genes (*slr1511*, *sll1069*, *slr1332* and *slr2023*), leading to a significant increase of malonyl-CoA content in cells (Fig. 7b, c). The results provided a new strategy for increasing malonyl-CoA in cyanobacterial chassis for producing malonyl-CoA-derived chemicals. In addition, a recent study demonstrated the importance of the fine-tuning for gene expression, in which the highest production of putrescine in *E. coli* was not observed in strains with the greatest knockdown of the *argF* gene expression, as the over downregulation of *argF* could result in growth inhibition [44]. Consistently, though *slr1511* and *slr2023* were almost fully blocked in WT-HMMA1, the enhancement of malonyl-CoA was not significant as the growth of WT-HMMA1 was also affected. On the contrary, though the four genes in WT-HMMA2 were only partially downregulated, a more significant increase of malonyl-CoA content was observed. Moreover, when modification of WT-HMMA1 to WT-HMMA1(the*) to allow biomass accumulation at the early stage was adopted, a higher increase (20%) on malonyl-CoA was achieved (Fig. 8c), suggesting the importance of fine-tuning and potential value of sRNA regulatory tools.

Recently, Gordon et al. [40] modified the CRISPR/dCas9 systems in *Synechococcus* sp. PCC 7002 by introducing the TetR repressor and the aTc-responsive promoter to control the dCas9 expression. Combined with weak RBS for dCas9 and a weak promoter for both sgRNA and dCas9, the modified system achieved a finely tuning regulation of the target gene with a downregulated efficiency up to 90% [40]. Nevertheless, this modified system still carried a ~ 30% leakage without induction [40]. The synthetic theophylline-dependent riboswitch has been screened and optimized in the early studies [48, 49]. A modified theophylline-dependent riboswitch was first introduced into cyanobacterium *Synechococcus elongatus* PCC 7942 in 2013 [35]. The transcript with this riboswitch could expose or sequester the RBS with or without the ligand theophylline, respectively, resulting in a negligible leakage and a significant induction up to 190-fold [35]. In this study, the theophylline-dependent riboswitch was utilized to control the expression of the chaperone protein Hfq and achieve a good performance of Hfq-MicC tool after induction, even though, ~ 20–30% leakage (depending on the binding energy between sRNA and its target) was still observed as the artificial sRNAs with 24 nucleotides complementary to the target mRNA could act as an antisense RNA. By fusing the theophylline-sensing aptamer to a spacer element followed by an aptamer-complementary sequence, the transcription-regulating riboswitch has been found working in *E. coli* [50]. To decrease the leakage, transcriptional regulating riboswitch could be utilized in the future to accurately control the expression of sRNAs.

## Conclusion

In this study, two small RNA regulatory tools are reported for *Synechocystis* based on PTRNAs and Hfq-MicC previously developed in *E. coli*. The results demonstrated that both regulatory tools were well functioning in *Synechocystis*. In addition, the Hfq-MicC tool was further developed to simultaneously regulate multiple genes related to essential fatty acids biosynthesis and generation of the key precursor malonyl-CoA. Moreover, the Hfq-MicC system was further modified to be an inducible system based on the theophylline-inducible riboswitch, achieving an optimized sRNA tool with good induction capability and relatively low leakage. The work introduces stable and efficient metabolic regulation strategies for photosynthetic cyanobacteria.

## Methods

### Bacterial growth conditions

WT and all *Synechocystis* construction strains were grown on BG-11 agar plate or in medium (pH 7.5) under a light intensity of approximately 50 μmoL photons m^−2^ s^−1^ in an illuminating shaking incubator (HNY-211B, Honour, Tianjin, China) at 130 rpm at 30 °C or incubator (SPX-250B-G, Boxun, Shanghai, China) at 30 °C, respectively [51]. Media for constructed strains were supplemented with appropriate antibiotic(s) when necessary (*i.e.*, 10 μg/mL chloramphenicol or/and 10 μg/mL spectinomycin). *E. coli* strains were grown on LB agar plate or in LB medium with appropriate antibiotic(s) to maintain plasmids (*i.e.*, 30 μg/mL chloramphenicol or 100 μg/mL spectinomycin) at 37 °C using incubator or shaking incubator (HNY-100B, Honour, Tianjin, China) at 200 rpm, respectively. Cell growth of *Synechocystis* was measured at OD_730 nm_ using a UV-1750 spectrophotometer (Shimadzu, Kyoto, Japan).

### Strain construction

*E. coli* DH5α strain was used for vector amplification and construction generation. All the primers were synthesized by GENEWIZ Inc. (Suzhou, China). Primers used in this study were listed in Additional file 2: Table S1 and the related sequences of cassettes used in this study were listed in Additional file 2: Table S2. All the strains constructed in this study were listed in Table 1. Transformation of *Synechocystis* was performed by electroporation (~ 10 μg plasmid DNA) using GenePulser Xcell (Bio-Rad, CA, USA) and grown photo-autotrophically on agar plate with 10 μg/mL spectinomycin and/or 10 μg/mL chloramphenicol [24]. All transformants were validated by colony PCR.

### *β*-Galactosidase activity

A total of 1 mL of *Synechocystis* (OD_730 nm_ = 1) culture were collected via centrifugation at 13,000×*g* and re-suspended using 1 mL Z Buffer (60 mM Na_2_HPO_4_, 40 mM NaH_2_PO_4_, 10 mM KCl, 1 mM MgSO_4_ and 40 mM *β*-mercaptoethanol). 50 μL 0.1% SDS, 50 μL chloroform, and 200 μL ortho-nitrophenyl-beta-d-galactopyranoside (ONPG; 4 mg/mL) were then added sequentially. The mixing solution was incubated at 30 °C for 20 min. Finally, 500 μL 1 M Na_2_CO_3_ was added to stop the reaction. The final solution was centrifuged for 2 min at 13,000×*g* and the supernatant was measured at OD_420 nm_ using the UV-1750 spectrophotometer (Shimadzu, Kyoto, Japan). The results were normalized using the absorption of WT at OD_420 nm_. LacZ activity was quantized using the formula “Miller = 1000 × OD_420 nm_/(1 mL × 20 min × OD_730 nm_)” according to a previous publication [52].

### Glycogen quantitation assays

Glycogen quantitation assays were performed using a glycogen quantitation Kit (Tiangen, Beijing, China) and modified based on the previous studies [32, 53]. Briefly, 1 mL fresh cultures of WT, WT-C, WT-HMC, WT-PTGLGC, and WT-HMGLGC (Volume*OD_730 nm_ = 1) were collected at 72 h by centrifugation at 14,000×*g* for 2 min. Then, the sample pellets were collected and re-suspended with 500 μL KOH solution (30%; *w/v*), and then incubated for 2 h at 95 °C. After that, 1500 μL of absolute ethanol was added into the sample and incubated for 2 h on ice. The sample was centrifuged at 14,000×*g* for 10 min and the precipitate was re-suspended using 500 μL of ddH_2_O and then incubated at 95 °C for 15 min. The sample was then cooled on ice and added with anthrone/H_2_SO_4_ solution (*w/v* = 2:1) and incubated at 95 °C for 15 min. The final solution was cooled on ice and measured at OD_625 nm_. The quantitation of glycogen was performed by a standard curve using a series concentration of glucose solution (Additional file 1: Fig. S3). The absorption at OD_625 nm_ by 111 μg glycogen was equal to that by 100 μg glucose according to the protocol.

### Nitrogen depletion assay

BG-11 medium without sodium nitrate (BG-N^−^) was used for the nitrogen depletion experiments [54]. Briefly, 5 mL fresh cultures of WT, WT-PTGLGC, and WT-HMGLGC (OD_730 nm_ = 0.5) were collected by centrifugation at 3000×*g* and 4 °C, washed twice using BG-N^−^ medium, and then inoculated into 25 mL of BG-N^−^ liquid medium in 100 mL flasks. The assays were performed both in an illuminating shaking incubator or incubator for a shaking or static culture and repeated for 3 times to ensure the phenotype.

### qRT-PCR analysis

Total RNA extraction was achieved through a Direct-zol™ RNA MiniPrep Kit (Zymo, CA, USA). *Synechocystis* samples were collected at 48 h. Approximately 10 mL of samples (OD_730 nm_ = 0.5) were collected by centrifugation (7000×*g* for 10 min), frozen immediately by liquid nitrogen after centrifugation, and subjected to RNA extraction following the manufacturer’s protocol. cDNAs were synthesized using RevertAid First Strand cDNA Synthesis Kit following the manufacturer’s protocol (Thermo Fisher Scientific Inc., CA, USA), and then, 1 μL of each dilution was used as template for following qRT-PCR reaction. The qPCR reaction was carried out in 10 μL reactions containing 5 μL of UltraSYBR Mixture (CW Biotech, Beijing, China), 3 μL dH_2_O, 1 μL diluted template cDNA, and 1 μL of each PCR primer, employing the StepOnePlus™ Real-Time PCR System (Applied Biosystems, CA, USA) [24]. Three technical replicates were performed for each condition. Data analysis was carried out using the StepOnePlus analytical software (Applied Biosystems, CA, USA) and the 2^−ΔΔCT^ method [55]. 16S rRNA was selected as a reference gene and the data were presented as ratios of the amount of normalized transcript in the constructed strain to that from the WT control [24].

### RT-PCR analysis

The RNA extraction and cDNA synthesis were the same as described above. Then, specific forward primers of each sRNAs and the reverse primer micC-R were used to amplify each sRNAs (Primers were listed in Additional file 2: Table S1). 16S rRNA was selected as a reference.

### Lipid profiles

Total lipids were extracted from approximate 5 mg dry cells of the WT and WT-HMFA strain in 2 mL chloroform/methanol (*v*/*v*, 2:1) (each with 3 biological replicates) using ultrasonic treatment for 10 min and centrifugation at 7000×*g* for 5 min. Then, 5 mL 2.0% H_2_SO_4_–methanol (*v*/*v*, H_2_SO_4_/methanol) was added into the collected supernatants, and the flask was stirred at 70 °C for 1 h. After 2 mL of hexane and 0.75 mL of distilled water were added to the flask and mixed, the hexane layer contained the fatty acid methyl esters (FAMEs) was transferred to a new vial and mixed with the internal standard C17-ME for gas chromatography (GC) analyses. FAME analyses were carried out by an Agilent 6890 GC instrument (CA, USA) and FAME yield was calculated using the equation described previously by Liu et al. [56].

### Malonyl-CoA quantitation

Quantitation of malonyl-CoA was performed using a “Bacterial Malonyl-CoA ELISA Kit” following the protocols (Dongge Biotech Inc., Beijing, China). Briefly, 1 mL of *Synechocystis* (OD_730 nm_ = 0.5) culture was collected by centrifugation at 4 °C (13,000×*g*, 2 min). Cell pellets were re-suspended using 50 μL ddH_2_O and then crushed by multi-gelation with liquid nitrogen. After that, 10 μL supernatant and 40 μL diluent buffer were added into a 96-well plate. Then, 50 μL of HRP-conjugate and 50 μL of antibody were added into each well. The mixture was incubated under 37 °C for 1 h and then washed five times using the wash buffer. After that, 50 μL of Substrate A and 50 μL of Substrate B were added and then incubated for 15 min at 37 °C. Finally, the reaction was stopped by adding 50 μL stop solution and measured at OD_450 nm_ using the ELx808 Absorbance Microplate Reader (BioTek, Winooski, VT, USA). The standard curve was plotted using different dilutions of malonyl-CoA standard (Dongge Biotech Inc., Beijing, China) (Additional file 1: Fig. S1).

### Theophylline treatment

The stock solution of theophylline was prepared by dissolving 10 mmol theophylline (Aladdin; Shanghai; China) in 1 L normal BG11 medium. For theophylline-induced assays, *Synechocystis* samples were collected by centrifugation at 3000×*g* and 4 °C for 15 min and then re-suspended using fresh BG11 medium and corresponding amount of the stock solution of theophylline.

### **SDS-PAGE**

SDS-PAGE was performed for WT, un-induced WT-HMMA1(the*), and 2 mM theophylline-induced WT-HMMA1(the*). Briefly, samples (volume*OD_730 nm_ = 5) were collected by centrifugation at 7500×*g* and 4 °C for 10 min. Then, the cell pellets were collected and re-suspended using 100 μL ddH_2_O. The samples were then crushed by multi-gelation with liquid nitrogen, and after another centrifugation (14,000×*g* for 2 min), supernatant was then used for SDS-PAGE. The total protein was quantified using the NanoDrop™ 2000/2000c spectrophotometers (Thermo Fisher Scientific Inc., CA, USA), separated on 15% SDS–polyacrylamide gel and stained by coomassie blue [57, 58].

## Additional files

**Additional file 1: Figure S1.** Schematic of the constructed vectors. Detailed sequences were available in Additional file 2: Table S2. A) Schematic of the pCP0168 and pBA0168. B) Schematic of the pBA3031M. **Fig. S2.** Validation of the expression of sRNAs in constructed strains using RT-PCR. A) The expression of sRNAs in WT-HMC, WT-PTGLGC, WT-HMGLGC, WT-HMFA, WT-HMMA1, WT-HMMA2, and WT-HMMA1(the*). Lane 1–12: 1. WT-HMC using micC-R and RT-GlgC-F; 2. WT-PTGLGC using AsGlgc1-F and RT-PTGlgC-R; 3. WT-HMGLGC using micC-R and RT-GlgC-F; 4. WT-HMFA using micC-R and RT-sll1069-F; 5. WT-HMFA using micC-R and RT-slr1332-F; 6. WT-HMFA using micC-R and RT-slr1511-F; 7. WT-HMMA1 using micC-R and RT-slr1511-F; 8. WT-HMMA1 using micC-R and RT-slr2023-F; 9. WT-HMMA2 using micC-R and R RT-sll1069-F; 10. WT-HMMA2 using micC-R and RT-slr1332-F; 11. WT-HMMA2 using micC-R and RT-slr1511-F; 12. WT-HMMA2 using micC-R and RT-slr2023-F. B) The expression of sRNAs in WT-HMFA, WT-HMMA1, and WT-HMMA2. Lane 1–12: 1. WT-HMFA using micC-R and RT-sll1069-F; 2. WT-HMFA using micC-R and RT-slr1332-F; 3. WT-HMFA using micC-R and RT-slr1511-F; 4. WT-HMMA1 using micC-R and RT-slr1511-F; 5. WT-HMMA1 using micC-R and RT-slr2023-F; 6. WT-HMMA2 using micC-R and R RT-sll1069-F; 7. WT-HMMA2 using micC-R and RT-slr1332-F; 8. WT-HMMA2 using micC-R and RT-slr1511-F; 9. WT-HMMA2 using micC-R and RT-slr2023-F; 10. HMFA using qRT-16S-F and qRT-16S-R; 11. HMMA1 using qRT-16S-F and qRT-16S-R; 12. HMMA2 using qRT-16S-F and qRT-16S-R. **Fig. S3.** Standard curve for glycogen measurement. A series of glucose standard diluents were utilized. **Fig. S4.** Standard curve for malonyl-CoA measurement. A series of malonyl-CoA standard diluents were utilized. **Fig. S5.** Bleaching phenotype of HMMA1 compared to WT. **Fig. S6.** A) Schematic of the theophylline-inducible riboswitch. B) Schematic of the pBA3031-HM(the*). **Fig. S7.** The dose effect of the theophylline on the growth of WT. The error bar represents the standard deviation of the three biological replicates for each sample. **Fig. S8.** SDS-PAGE for WT and WT-HMMA1(the*) with or without induction. Lane 1–7: 1 was WT; 2–4 were WT-HMMA1(the*) without induction and 5–7 were WT-HMMA1(the*) with induction. The Hfq was shown using red arrows. **Fig. S9.** Binding energies distributions of all the genes of *Synechocystis* genome.

**Additional file 2: Table S1.** All the primers used in this study. **Table S2.** Detailed sequences for constructed plasmids in this study. **Table S3.** Sequences used for calculating binding energies between sRNAs and their corresponding targets.

## Abbreviations

CRISPR/Cas system: clustered regularly interspaced short palindromic repeats/CRISPR-associated proteins
FAMEs: fatty acid methyl esters
GC: gas chromatography
3-HP: 3-hydroxypropionic acid
PTRNAs: paired termini RNAs
sRNA: small RNA
RBS: ribosome-binding site
WT: wild type
