# Supplementary material for: Re-direction of carbon flux to key precursor malonyl-CoA via artificial small RNAs in photosynthetic Synechocystis sp. PCC 6803
Source: Biotechnol Biofuels. 2018 Feb 5;11:26. doi: 10.1186/s13068-018-1032-0 (PMC5798194; doi:10.1186/s13068-018-1032-0)
Supplement: Supplementary file 1 — Additional file 1: Figure S1. Schematic of the constructed vectors. Detailed sequences were available in Additional file 2: Table S2. A) Schematic of the pCP0168 and pBA0168. B) Schematic of the pBA3031M. Fig. S2. Validation of the expression of sRNAs in constructed strains using RT-PCR. A) The expression of sRNAs in WT-HMC, WT-PTGLGC, WT-HMGLGC, WT-HMFA, WT-HMMA1, WT-HMMA2, and WT-HMMA1(the*). Lane 1–12: 1. WT-HMC using micC-R and RT-GlgC-F; 2. WT-PTGLGC using AsGlgc1-F and RT-PTGlgC-R; 3. WT-HMGLGC using micC-R and RT-GlgC-F; 4. WT-HMFA using micC-R and RT-sll1069-F; 5. WT-HMFA using micC-R and RT-slr1332-F; 6. WT-HMFA using micC-R and RT-slr1511-F; 7. WT-HMMA1 using micC-R and RT-slr1511-F; 8. WT-HMMA1 using micC-R and RT-slr2023-F; 9. WT-HMMA2 using micC-R and R RT-sll1069-F; 10. WT-HMMA2 using micC-R and RT-slr1332-F; 11. WT-HMMA2 using micC-R and RT-slr1511-F; 12. WT-HMMA2 using micC-R and RT-slr2023-F. B) The expression of sRNAs in WT-HMFA, WT-HMMA1, and WT-HMMA2. Lane 1–12: 1. WT-HMFA using micC-R and RT-sll1069-F; 2. WT-HMFA using micC-R and RT-slr1332-F; 3. WT-HMFA using micC-R and RT-slr1511-F; 4. WT-HMMA1 using micC-R and RT-slr1511-F; 5. WT-HMMA1 using micC-R and RT-slr2023-F; 6. WT-HMMA2 using micC-R and R RT-sll1069-F; 7. WT-HMMA2 using micC-R and RT-slr1332-F; 8. WT-HMMA2 using micC-R and RT-slr1511-F; 9. WT-HMMA2 using micC-R and RT-slr2023-F; 10. HMFA using qRT-16S-F and qRT-16S-R; 11. HMMA1 using qRT-16S-F and qRT-16S-R; 12. HMMA2 using qRT-16S-F and qRT-16S-R. Fig. S3. Standard curve for glycogen measurement. A series of glucose standard diluents were utilized. Fig. S4. Standard curve for malonyl-CoA measurement. A series of malonyl-CoA standard diluents were utilized. Fig. S5. Bleaching phenotype of HMMA1 compared to WT. Fig. S6. A) Schematic of the theophylline-inducible riboswitch. B) Schematic of the pBA3031-HM(the*). Fig. S7. The dose effect of the theophylline on the growth of WT. The error bar represents the standard deviation of the three [file 13068_2018_1032_MOESM1_ESM.pptx]

## Slide 1
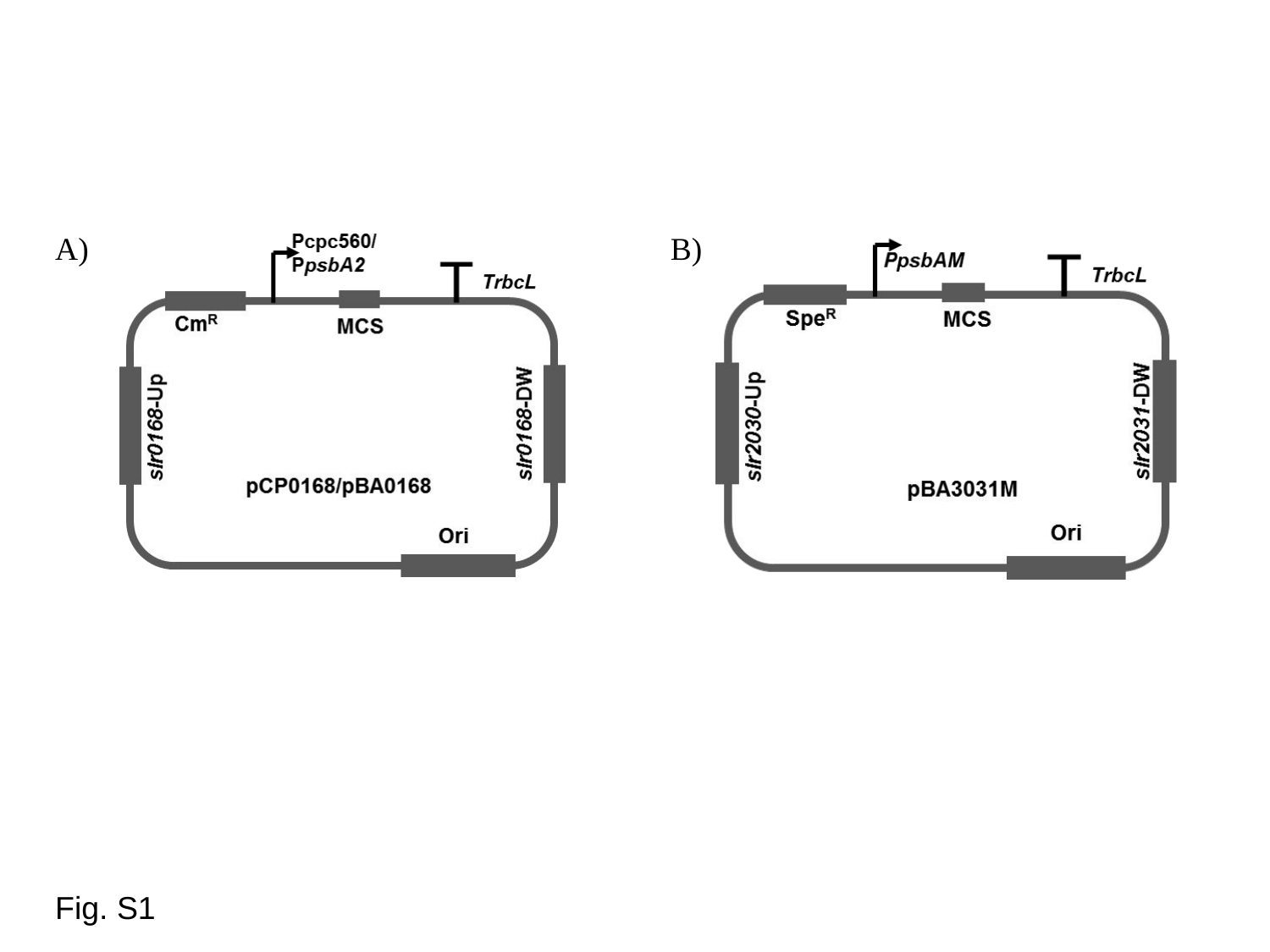

A)
B)
Fig. S1

## Slide 2
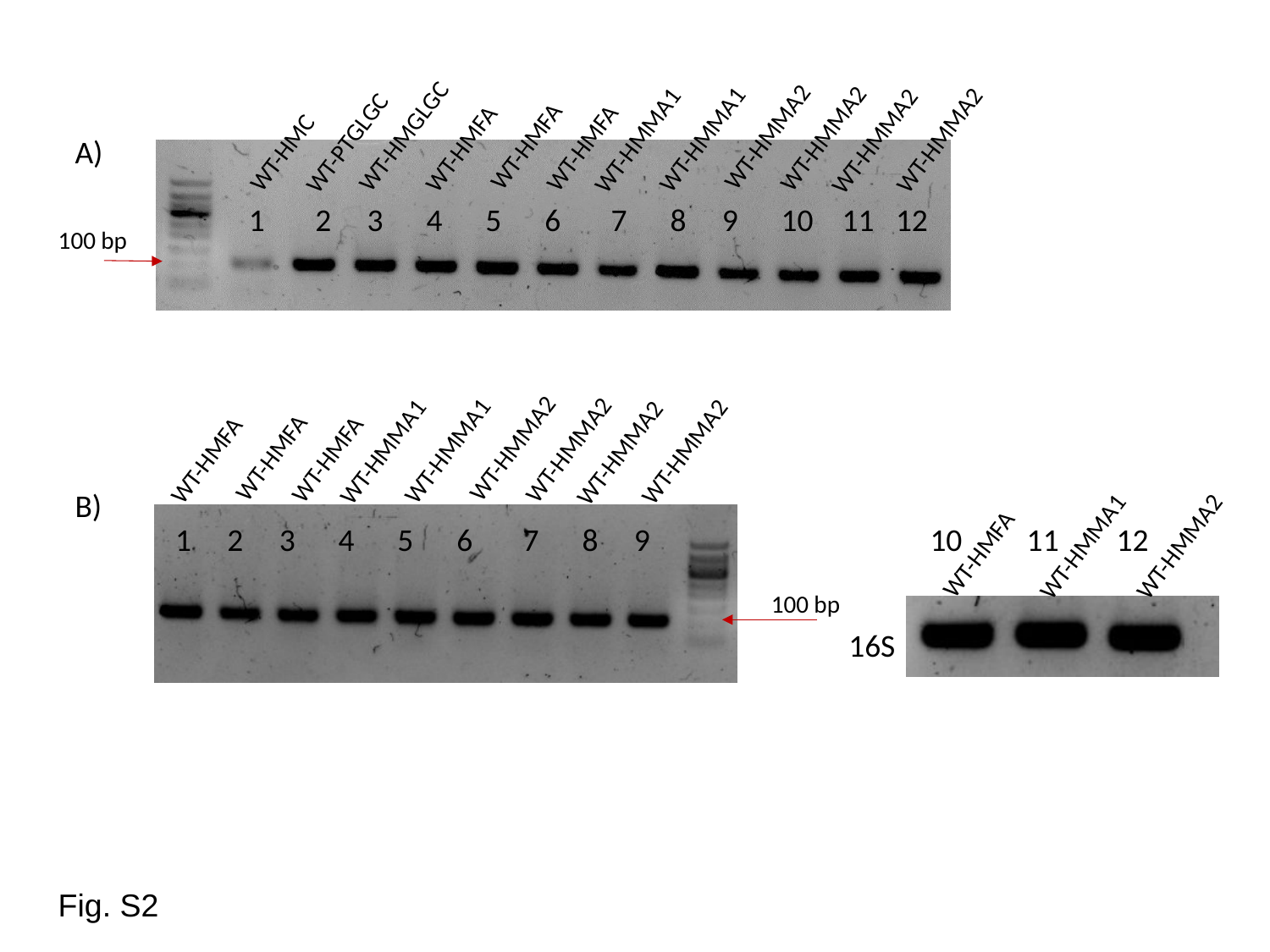

WT-HMGLGC
WT-HMFA
WT-HMMA2
WT-HMFA
WT-HMMA2
WT-HMFA
WT-HMMA1
WT-PTGLGC
WT-HMC
WT-HMMA2
WT-HMMA1
WT-HMMA2
A)
1 2 3 4 5 6 7 8 9 10 11 12
100 bp
WT-HMFA
WT-HMMA2
WT-HMFA
WT-HMMA2
WT-HMFA
WT-HMMA1
WT-HMMA2
WT-HMMA1
WT-HMMA2
B)
1 2 3 4 5 6 7 8 9 10 11 12
WT-HMFA
WT-HMMA2
WT-HMMA1
100 bp
16S
Fig. S2

## Slide 3
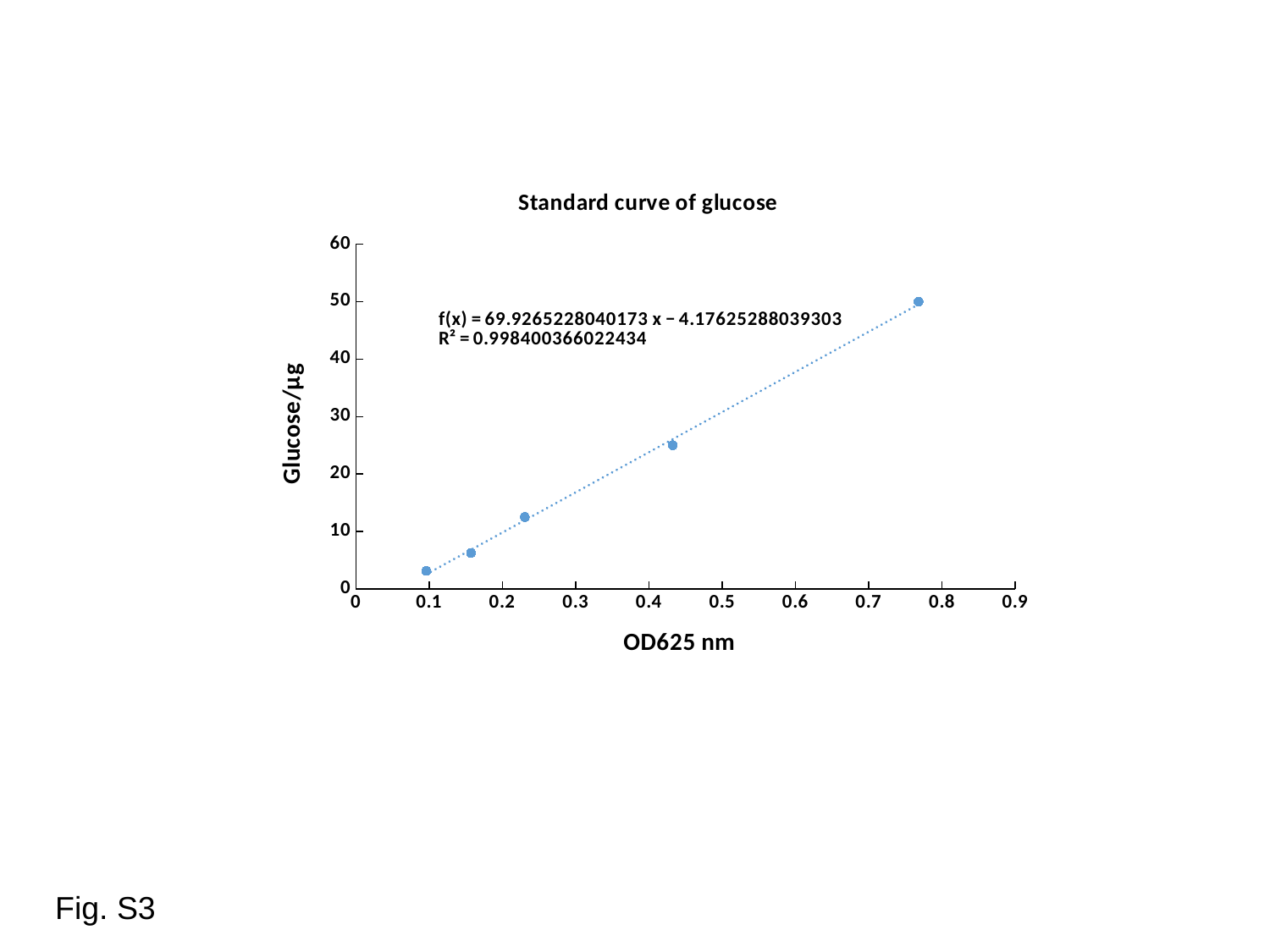

### Chart: Standard curve of glucose
| Category | |
|---|---|Fig. S3

## Slide 4
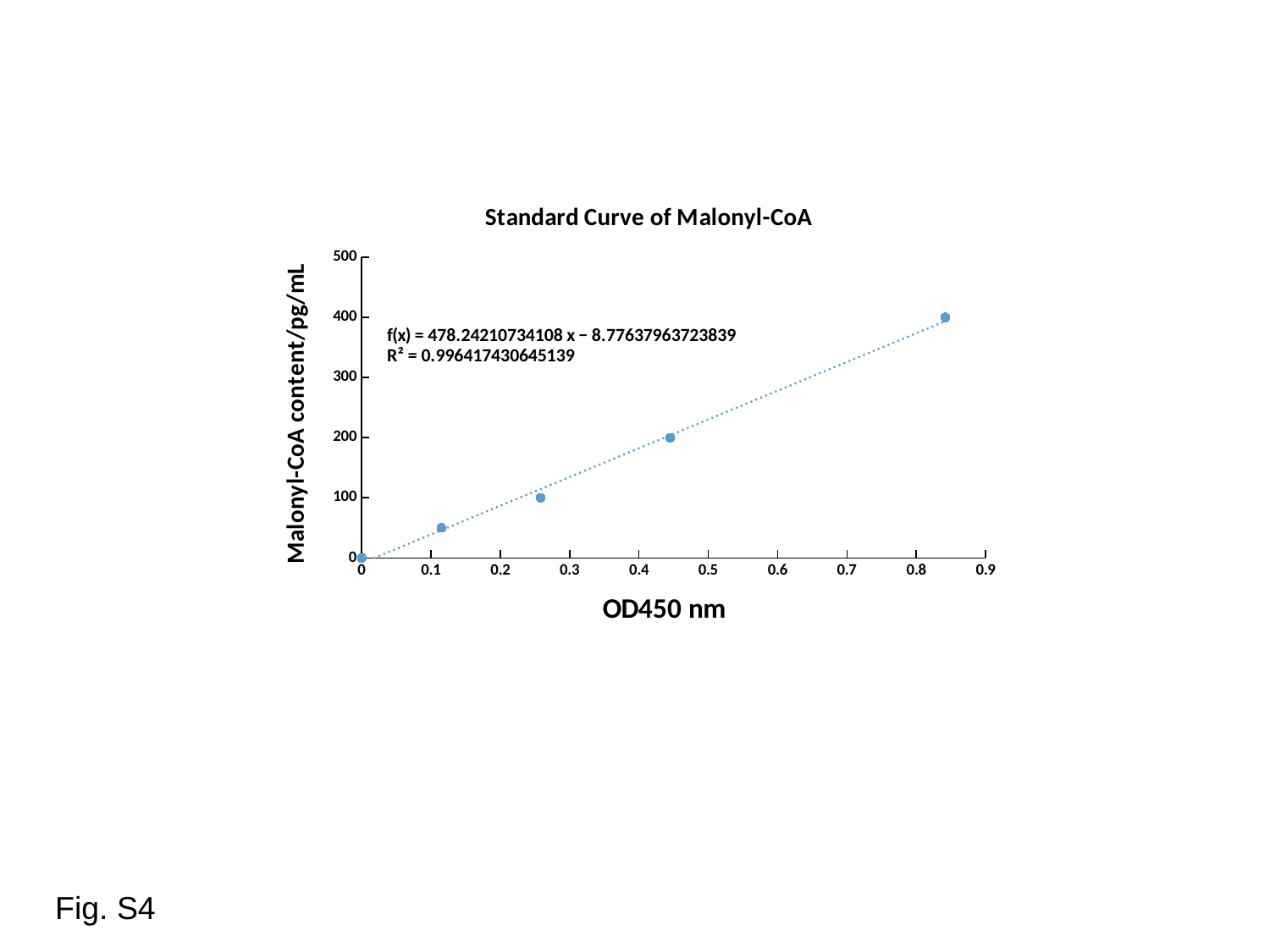

### Chart: Standard Curve of Malonyl-CoA
| Category | |
|---|---|Fig. S4

## Slide 5
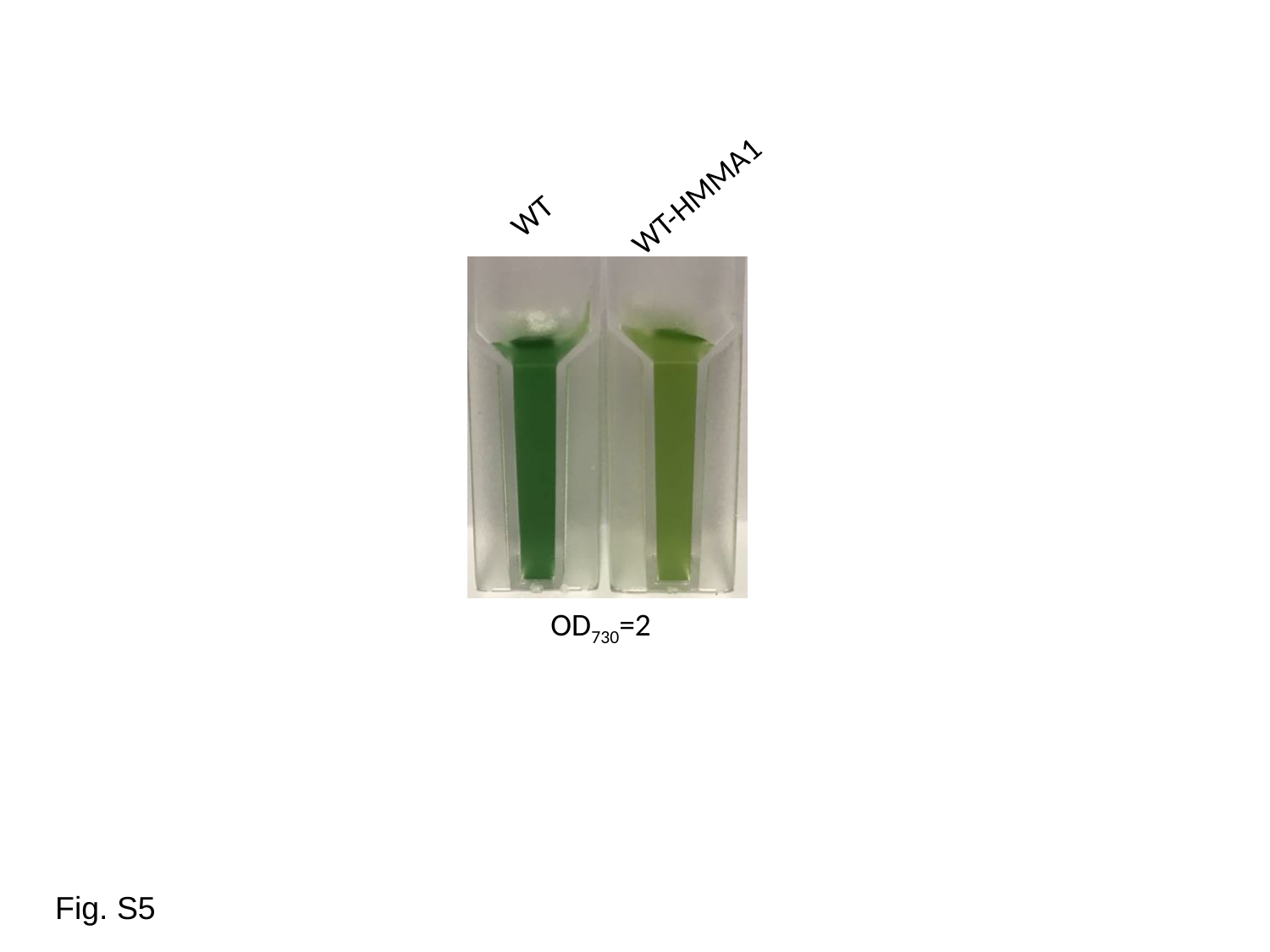

WT-HMMA1
WT
OD730=2
Fig. S5

## Slide 6
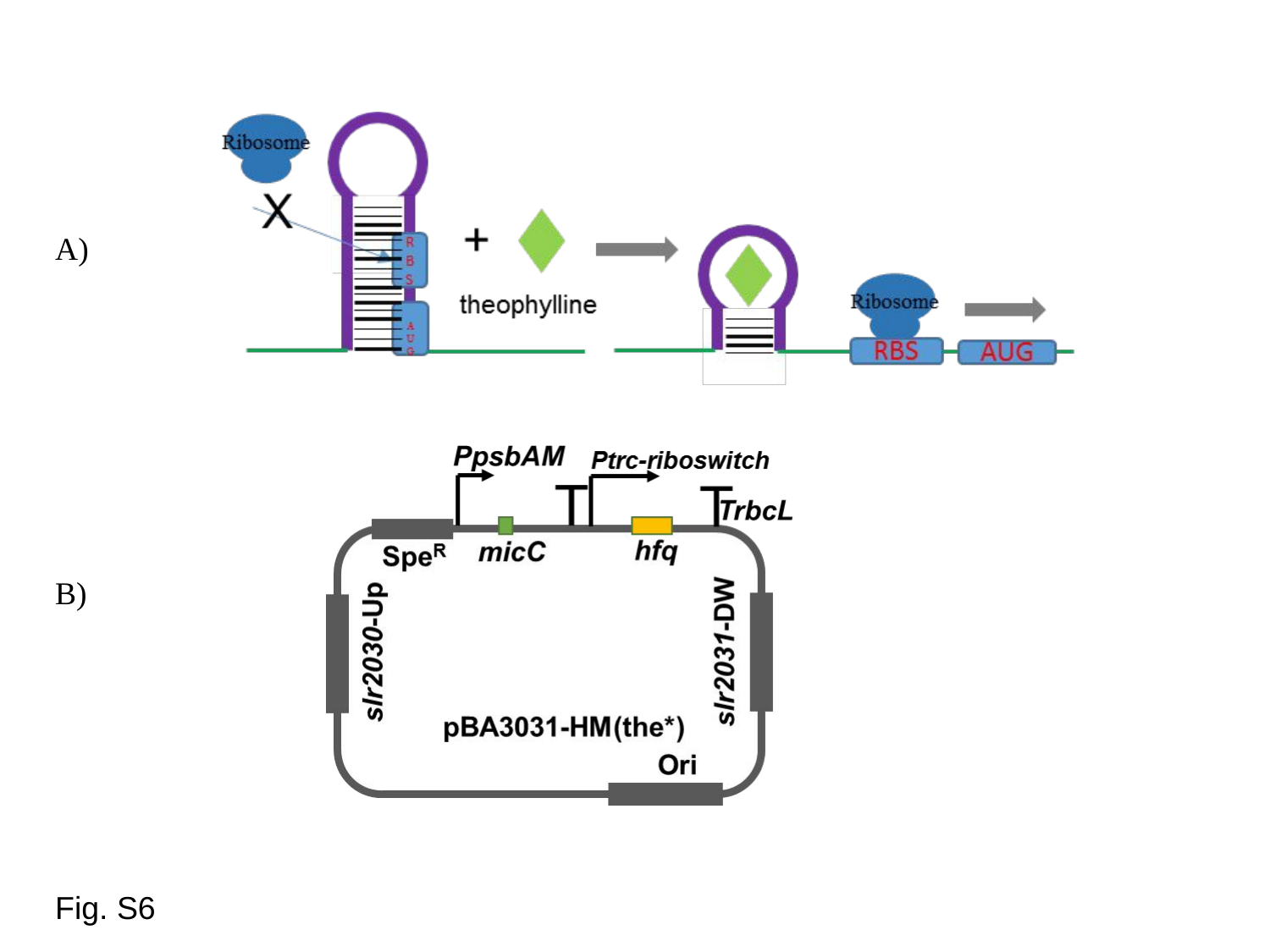

A)
B)
Fig. S6

## Slide 7
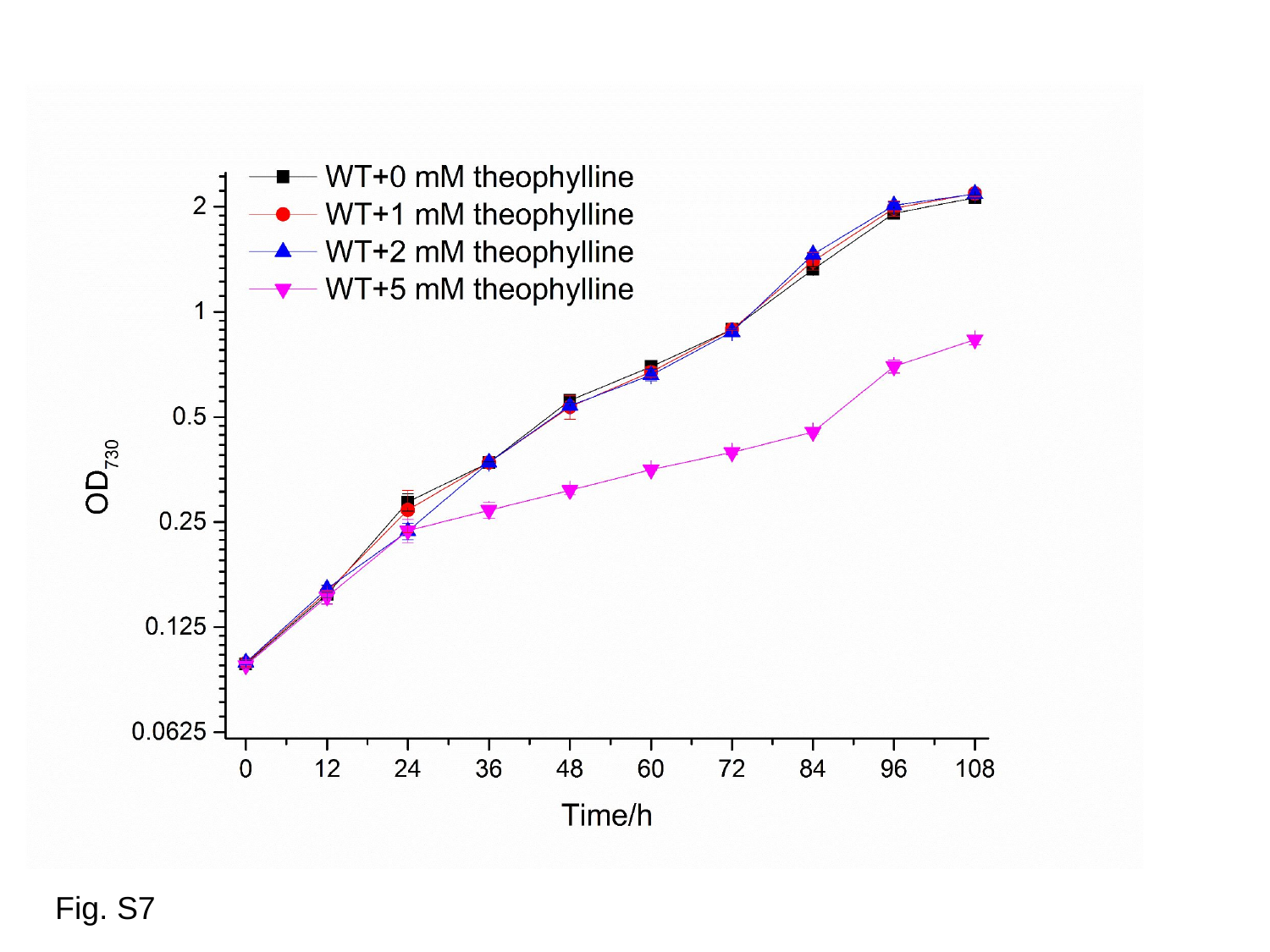

Fig. S7

## Slide 8
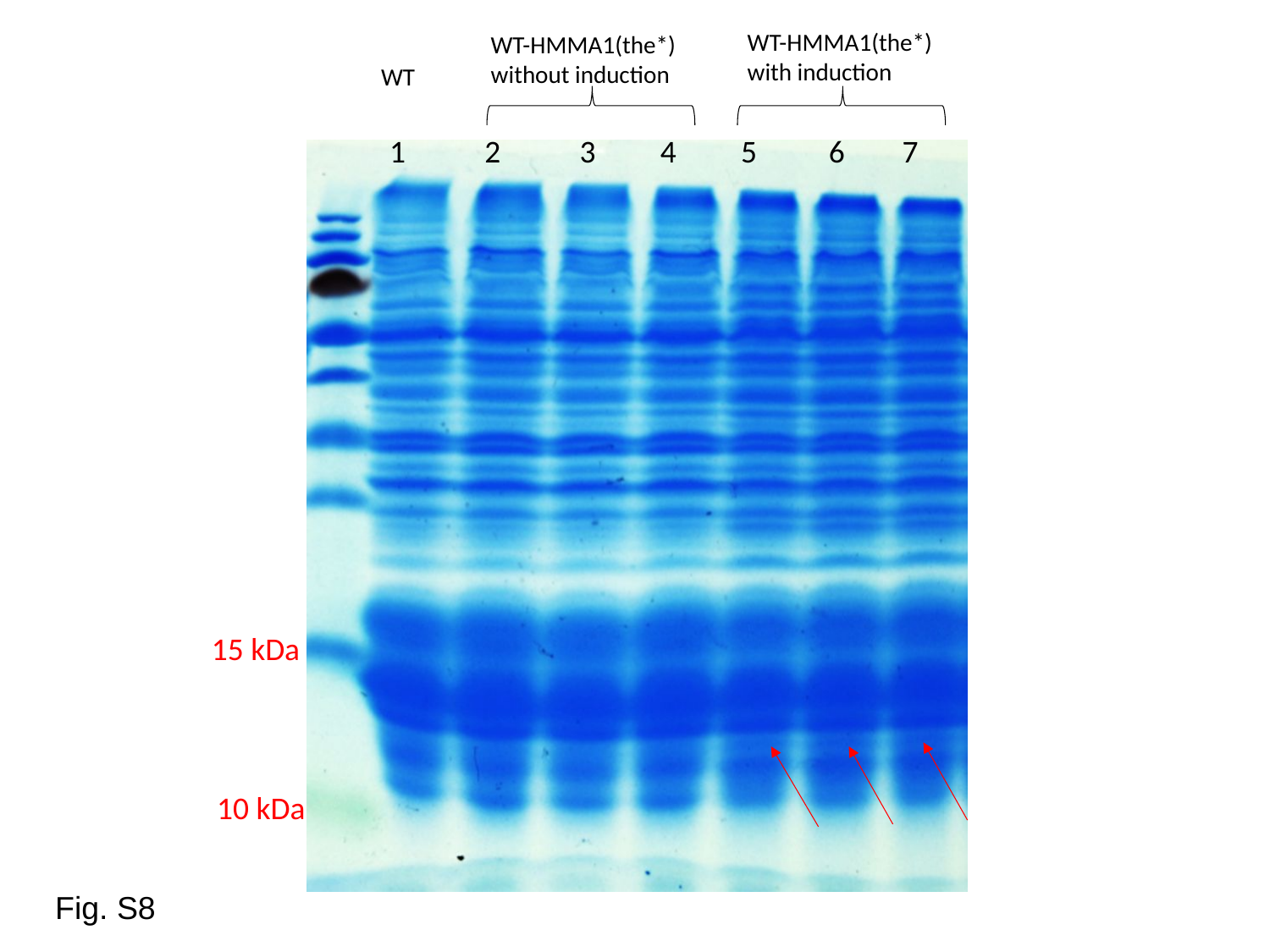

WT-HMMA1(the*)
with induction
WT-HMMA1(the*)
without induction
WT
1 2 3 4 5 6 7
15 kDa
10 kDa
Fig. S8

## Slide 9
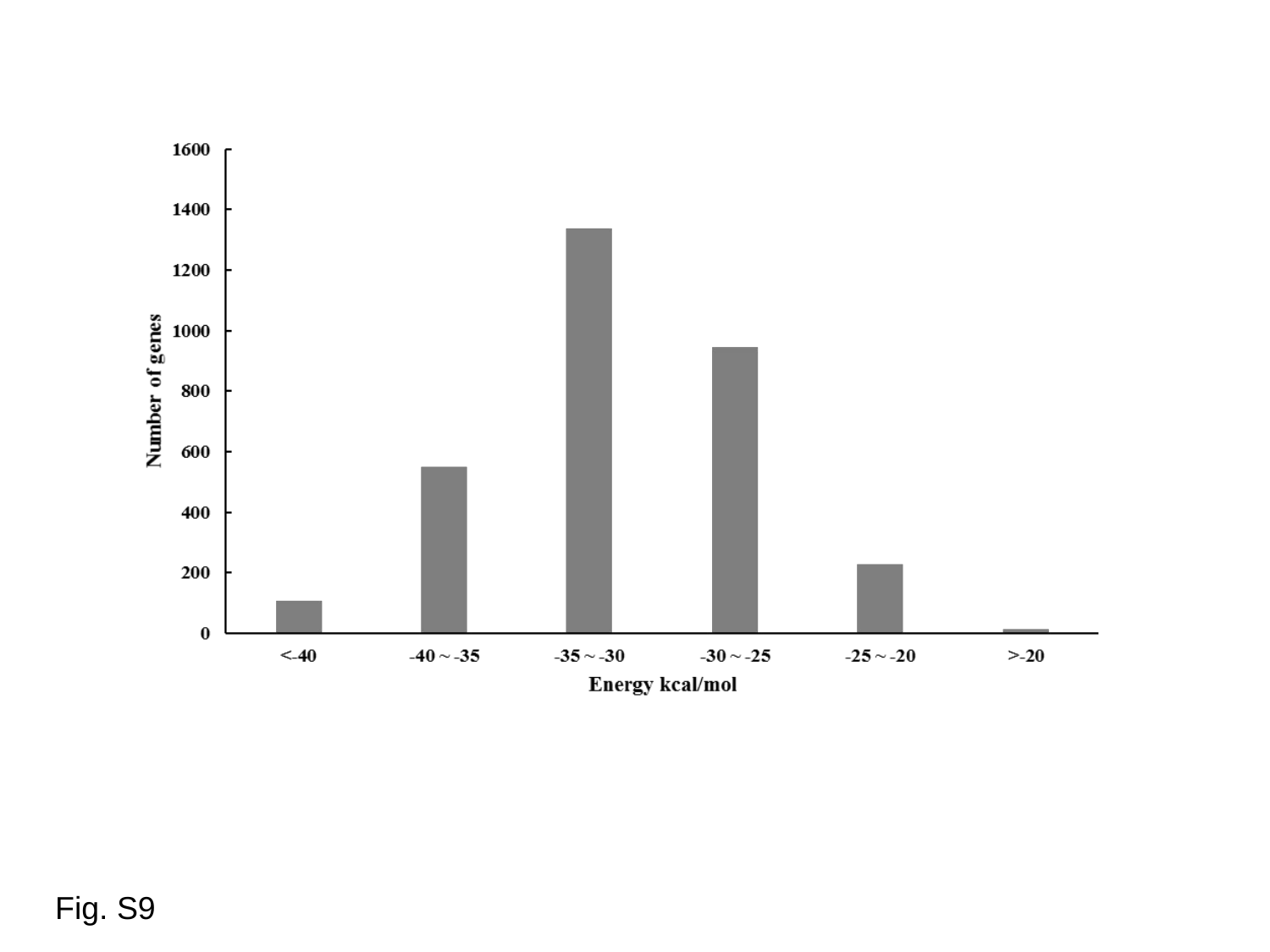

Fig. S9
