# Supplementary material for: Re-direction of carbon flux to key precursor malonyl-CoA via artificial small RNAs in photosynthetic Synechocystis sp. PCC 6803
Source: Biotechnol Biofuels. 2018 Feb 5;11:26. doi: 10.1186/s13068-018-1032-0 (PMC5798194; doi:10.1186/s13068-018-1032-0)
Supplement: Supplementary file 2 — Additional file 2: Table S1. All the primers used in this study. Table S2. Detailed sequences for constructed plasmids in this study. Table S3. Sequences used for calculating binding energies between sRNAs and their corresponding targets. [file 13068_2018_1032_MOESM2_ESM.docx]

**Table S1. All the primers used in this study.**

| Primers for strains construction | |
| --- | --- |
| Trbcl-F | ACCGGTGTTTGGATTGTCGG |
| Trbcl-R | CGGGATCCGCTGTCGAAGTTGAACATC |
| Ppsba2-F | TATCAATAAGTATTAGGTAT |
| Ppsba2M-R | ATGTATTTGTCGATGTTCAG |
| cpc560-F | ACCTGTAGAGAAGAGTCCCT |
| cpc560-R | TGAATTAATCTCCTACTTGA |
| KpnI-Spe-F | GGGGTACCCGCACACCGTGGAAACGGAT |
| EcoR-Spe-R | CGGAATTCTTATTTGCCGACTACCTTGG |
| HindIII-0168U-F | CCAAGCTTATTCCCGGTATGGATGGCAC |
| 0168U-T-R | TGGGCCTTTCTGCGTTTATAATTTTGATTGGTGGCTAACC |
| T-asCm-F | TCGTTTTATTTGATGCCTGGTTACGCCCCGCCCTGCCACT |
| SmaI-asCm-R | CCCCCGGGTACCTGTGACGGAAGATCAC |
| T0015-F | TATAAACGCAGAAAGGCCCA |
| T0015-R | CCAGGCATCAAATAAAACGAA |
| Cpc-F | ACCTGTAGAGAAGAGTCCCT |
| Cpc-mcs-R | AGGCCTCATATGGATATCAAGATCTTCTCGAGTGAATTAATCTCCTACTTGA |
| Kpn-Trbcl-F | GGGGTACCACCGGTGTTTGGATTGTCGG |
| Trbcl-R | GCTGTCGAAGTTGAACATCA |
| T-0168D-F | TGATGTTCAACTTCGACAGCAACAGTGGCGGGGATTTATA |
| EcoR-0168D-R | CGGAATTCATCTTTAACCGCTAAACCCA |
| Hind-2030U-F | CCAAGCTTGCTTTGCAAAAGGAATTTGC |
| 2030U-T-R | TGGGCCTTTCTGCGTTTATATTAGCAATGGCGGGAGACAG |
| T-asSpe-F | TCGTTTTATTTGATGCCTGGTTATTTGCCGACTACCTTGG |
| Sma-asSpe-R | CCCCCGGGCGCACACCGTGGAAACGGAT |
| T-2031D-F | TGATGTTCAACTTCGACAGCAAAACCCCGTTAATTTTTCT |
| EcoR-2031D-R | CGGAATTCACTCAAAATGGGGGTGTTAT |
| Xho-Hfq-F | CCCTCGAGATGGCTAAGGGGCAATCTTT |
| Bgl-Hfq-R | GAAGATCTTTATTCGGTTTCTTCGCTGT |
| MicC-F | TTTCTGTTGGGCCATTGCATTGCCACTGATTTTCCAACATATAAAAAGACAAGCCCGAACAGTCGTCCGGGCTTTTTTT |
| MicC-R | AAAAAAAGCCCGGACGACTGTTCGGGCTTGTCTTTTTATATGTTGGAAAATCAGTGGCAATGCAATGGCCCAACAGAAA |
| AsGlgC2-F | ACCTCTCGATTGCCAACAACACACTTTCTGTTGGGCCATTGCATT |
| AslacZ2-F | GACGGCCAGTGAATCCGTAATCATTTTCTGTTGGGCCATTGCATT |
| Asslr1511-micc-F | GAGGCCACTGCCAAGGGTATTCAATTTCTGTTGGGCCATTGCAT |
| Assll1069-micc-F | ACGTTTCTTTTCCAAATTTGCCATTTTCTGTTGGGCCATTGCAT |
| Asslr1332-micc-F | TTTTTGTTTCCCCTGGCGGTCCATTTTCTGTTGGGCCATTGCAT |
| Asslr2023-Micc-F | GGGGAATACCCATGCAGTTTTCATTTTCTGTTGGGCCATTGCAT |
| PT1-Ppasb-R | GCACCACCACCACCACCACTGCATGGTTAATTCCTCCTATGTATTTGTCGATGTTCAG |
| AslacZ1-F | TGGCGAAAGGGGGATGTGCT |
| Lacz-PT2-R | AGGAGGAATTAACCATGCAGTGGTGGTGGTGGTGGTGCATGATTACGGATTCACTGGC |
| AsGlgc1-F | TGAGTTTGGTTAAAGGATAG |
| Glgc-PT2-R | AGGAGGAATTAACCATGCAGTGGTGGTGGTGGTGGTGCGTGTGTTGTTGGCAATCGAG |
| Lacz-F | ATGATTACGGATTCACTGGC |
| Lacz-R | TTACTTCTGACACCAAACCA |
| **Primers for qRT-PCR** | |
| qRT-16S-F | TGTAGCGGTGAAATGCGTAG |
| qRT-16S-R | CCACGCCTAGTATCCATCGT |
| qRT-GlgC-F | CCAATGCCGACATAACCCTTT |
| qRT-GlgC-R | CCAGCTCGGGTGCCTTT |
| qRT-slr1511-F | CAGAGCCTTTGCCTTTGACAT |
| qRT-slr1511-R | CAACCCCACCACAAAACCA |
| qRT-sll1069-F | CGGGCATTGGTGGTTTGA |
| qRT-sll1069-R | TAGGACCCTTATCCAACAGAATGG |
| qRT-slr1332-F | GCTAGCACCGGATGGATGTTA |
| qRT-slr1332-R | CCCCTTCCCCCAACACTAA |
| qRT-slr2023-F | CGAGCCGTGCCTCTGAAG |
| qRT-slr2023-R | GGAAGGTTGGGCCATAAAGC |
| **Primers for RT-PCR** | |
| RT-GlgC-F | ACCTCTCGATTGCCAACAACACAC |
| RT-slr1511-F | GAGGCCACTGCCAAGGGTATTCAA |
| RT-sll1069-F | ACGTTTCTTTTCCAAATTTGCCAT |
| RT-slr1332-F | TTTTTGTTTCCCCTGGCGGTCCAT |
| RT-slr2023-F | GGGGAATACCCATGCAGTTTTCAT |
| RT-PTGlgC-R | GTGTGTTGTTGGCAATCGAG |
| micC-R | AAAAAAAGCCCGGACGACTGTTCG |

**Table S2. Detailed sequences for constructed plasmids in this study.**

| >pTZ57R/T backbone | ATCGGATCCCGGGCCCGTCGACTGCAGAGGCCTGCATGCAAGCTTTCCCTATAGTGAGTCGTATTAGAGCTTGGCGTAATCATGGTCATAGCTGTTTCCTGTGTGAAATTGTTATCCGCTCACAATTCCACACAACATACGAGCCGGAAGCATAAAGTGTAAAGCCTGGGGTGCCTAATGAGTGAGCTAACTCACATTAATTGCGTTGCGCTCACTGCCCGCTTTCCAGTCGGGAAACCTGTCGTGCCAGCTGCATTAATGAATCGGCCAACGCGCGGGGAGAGGCGGTTTGCGTATTGGGCGCTCTTCCGCTTCCTCGCTCACTGACTCGCTGCGCTCGGTCGTTCGGCTGCGGCGAGCGGTATCAGCTCACTCAAAGGCGGTAATACGGTTATCCACAGAATCAGGGGATAACGCAGGAAAGAACATGTGAGCAAAAGGCCAGCAAAAGGCCAGGAACCGTAAAAAGGCCGCGTTGCTGGCGTTTTTCCATAGGCTCCGCCCCCCTGACGAGCATCACAAAAATCGACGCTCAAGTCAGAGGTGGCGAAACCCGACAGGACTATAAAGATACCAGGCGTTTCCCCCTGGAAGCTCCCTCGTGCGCTCTCCTGTTCCGACCCTGCCGCTTACCGGATACCTGTCCGCCTTTCTCCCTTCGGGAAGCGTGGCGCTTTCTCATAGCTCACGCTGTAGGTATCTCAGTTCGGTGTAGGTCGTTCGCTCCAAGCTGGGCTGTGTGCACGAACCCCCCGTTCAGCCCGACCGCTGCGCCTTATCCGGTAACTATCGTCTTGAGTCCAACCCGGTAAGACACGACTTATCGCCACTGGCAGCAGCCACTGGTAACAGGATTAGCAGAGCGAGGTATGTAGGCGGTGCTACAGAGTTCTTGAAGTGGTGGCCTAACTACGGCTACACTAGAAGAACAGTATTTGGTATCTGCGCTCTGCTGAAGCCAGTTACCTTCGGAAAAAGAGTTGGTAGCTCTTGATCCGGCAAACAAACCACCGCTGGTAGCGGTGGTTTTTTTGTTTGCAAGCAGCAGATTACGCGCAGAAAAAAAGGATCTCAAGAAGATCCTTTGATCTTTTCTACGGGGTCTGACGCTCAGTGGAACGAAAACTCACGTTAAGGGATTTTGGTCATGAGATTATCAAAAAGGATCTTCACCTAGATCCTTTTAAATTAAAAATGAAGTTTTAAATCAATCTAAAGTATATATGAGTAAACTTGGTCTGACAGTTACCAATGCTTAATCAGTGAGGCACCTATCTCAGCGATCTGTCTATTTCGTTCATCCATAGTTGCCTGACTCCCCGTCGTGTAGATAACTACGATACGGGAGGGCTTACCATCTGGCCCCAGTGCTGCAATGATACCGCGAGACCCACGCTCACCGGCTCCAGATTTATCAGCAATAAACCAGCCAGCCGGAAGGGCCGAGCGCAGAAGTGGTCCTGCAACTTTATCCGCCTCCATCCAGTCTATTAATTGTTGCCGGGAAGCTAGAGTAAGTAGTTCGCCAGTTAATAGTTTGCGCAACGTTGTTGCCATTGCTACAGGCATCGTGGTGTCACGCTCGTCGTTTGGTATGGCTTCATTCAGCTCCGGTTCCCAACGATCAAGGCGAGTTACATGATCCCCCATGTTGTGCAAAAAAGCGGTTAGCTCCTTCGGTCCTCCGATCGTTGTCAGAAGTAAGTTGGCCGCAGTGTTATCACTCATGGTTATGGCAGCACTGCATAATTCTCTTACTGTCATGCCATCCGTAAGATGCTTTTCTGTGACTGGTGAGTACTCAACCAAGTCATTCTGAGAATAGTGTATGCGGCGACCGAGTTGCTCTTGCCCGGCGTCAATACGGGATAATACCGCGCCACATAGCAGAACTTTAAAAGTGCTCATCATTGGAAAACGTTCTTCGGGGCGAAAACTCTCAAGGATCTTACCGCTGTTGAGATCCAGTTCGATGTAACCCACTCGTGCACCCAACTGATCTTCAGCATCTTTTACTTTCACCAGCGTTTCTGGGTGAGCAAAAACAGGAAGGCAAAATGCCGCAAAAAAGGGAATAAGGGCGACACGGAAATGTTGAATACTCATACTCTTCCTTTTTCAATATTATTGAAGCATTTATCAGGGTTATTGTCTCATGAGCGGATACATATTTGAATGTATTTAGAAAAATAAACAAATAGGGGTTCCGCGCACATTTCCCCGAAAAGTGCCACCTGACGCGCCCTGTAGCGGCGCATTAAGCGCGGCGGGTGTGGTGGTTACGCGCAGCGTGACCGCTACACTTGCCAGCGCCCTAGCGCCCGCTCCTTTCGCTTTCTTCCCTTCCTTTCTCGCCACGTTCGCCGGCTTTCCCCGTCAAGCTCTAAATCGGGGGCTCCCTTTAGGGTTCCGATTTAGTGCTTTACGGCACCTCGACCCCAAAAAACTTGATTAGGGTGATGGTTCACGTAGTGGGCCATCGCCCTGATAGACGGTTTTTCGCCCTTTGACGTTGGAGTCCACGTTCTTTAATAGTGGACTCTTGTTCCAAACTGGAACAACACTCAACCCTATCTCGGTCTATTCTTTTGATTTATAAGGGATTTTGCCGATTTCGGCCTATTGGTTAAAAAATGAGCTGATTTAACAAAAATTTAACGCGAATTTTAACAAAATATTAACGCTTACAATTTCCATTCGCCATTCAGGCTGCGCAACTGTTGGGAAGGGCGATCGGTGCGGGCCTCTTCGCTATTACGCCAGCTGGCGAAAGGGGGATGTGCTGCAAGGCGATTAAGTTGGGTAACGCCAGGGTTTTCCCAGTCACGACGTTGTAAAACGACGGCCAGTGAATTCGAGCTCGGTACCTCGCGAATGCATCTAGATT |
| --- | --- |
| >upstream homologous arm of *slr0168* | ATTCCCGGTATGGATGGCACCGATGCGGAATCCCAACAGATTGCCTTTGACAACAATGTGGCCTGGAATAACCTGGGGGATTTGTCCACCACCACCCAACGGGCCTACACTTCGGCTATTAGCACAGACACAGTGCAGAGTGTTTATGGCGTTAATCTGGAAAAAAACGATAACATTCCCATTGTTTTTGCGTGGCCCATTTTTCCCACCACCCTTAATCCCACAGATTTTCAGGTAATGCTTAACACGGGGGAAATTGTCACCCCGGTGATCGCCTCTTTGATTCCCAACAGTGAATACAACGAACGGCAAACGGTAGTAATTACGGGCAATTTTGGTAATCGTTTAACCCCAGGCACGGAGGGAGCGATTTATCCCGTTTCCGTAGGCACAGTGTTGGACAGTACTCCTTTGGAAATGGTGGGACCCAACGGCCCGGTCAGTGCGGTGGGTATTACCATTGATAGTCTCAACCCCTACGTGGCCGGCAATGGTCCCAAAATTGTCGCCGCTAAGTTAGACCGCTTCAGTGACCTGGGGGAAGGGGCTCCCCTCTGGTTAGCCACCAATCAAAAT |
| >downstream homologous arm of *slr0168* | AACAGTGGCGGGGATTTATATGGAGACCAAGCCCAATTTCGTTTGCGAATTTACACCAGCGCCGGTTTTTCCCCCGATGGCATTGCCAGTTTACTACCCACAGAATTTGAACGGTATTTTCAACTCCAAGCGGAAGATATTACGGGACGGACAGTTATCCTAACCCAAACTGGTGTTGATTATGAAATTCCCGGCTTTGGTCTGGTGCAGGTGTTGGGGCTGGCGGATTTGGCCGGGGTTCAGGACAGCTATGACCTGACTTACATCGAAGATCATGACAACTATTACGACATTATCCTCAAAGGGGACGAAGCCGCAGTTCGCCAAATTAAGAGGGTTGCTTTGCCCTCCGAAGGGGATTATTCGGCGGTTTATAATCCCGGTGGCCCCGGCAATGATCCAGAGAATGGTCCCCCAGGGCCCTTTACTGTGTCCAGTAGTCCCCAGGTAATTAAGGTAACGGATACCATCGGCCAGCCCACCAAAGTCTCCTATGTGGAAGTGGATGGCCCCGTATTGCGTAATCCCTTCAGTGGTACTCCCATTGGGCAAGAGGTGGGTTTAGCGGTTAAAGAT |
| >upstream homologous arm of *slr2030* | GCTTTGCAAAAGGAATTTGCCCAGCAACTAGGTGGTAACTTTGTTTGGTTGGCCGATGAATGGTTTCTCATTGCCCGTCAACCTTTGCCACCGGAAAGCCATTACGAAGACTATCCCCAAATTGGCAACGGGGTAGGGTCTATCCGTCAATTCATTAAGGAGTTTCAACAACAAGCTGCGGAATTTCTCCCCCCGGCGATCGCCGAAGCCAAGACGTTGACCTGGGTAGTGGGCAATGCTGTAGAACAAGCGTTTGAGCTACTGGTGGAACAGTTAAATCAGGTAAAGGGTTTAACAGTTAATTTAGCGCCCTTAAACAGTGACTATTGGGGTCAGGAAATTACGGTGACGGGACTATTGACGGGGCAGGATTTAATCGCTAAATTGGCAGGCAGAGATTTAGGGGATGGTATTCTATTGCCTGCTCTGATGTTGAAACATGATGATACTCGCTTCTTGGATGACCTCCGGGTGGCCGATGTGGCTCAAAAGTTGGGCACAACCATTTACCCTGTGGCTGATGTGGCCAGTTTGTTGGAACATTGTGTTCAGCCCATGGCTGTCTCCCGCCATTGCTAA |
| >downstream homologous arm of *slr2031* | AAAACCCCGTTAATTTTTCTTACCCATGGGGAAGTTTGCTGGTTCAATAGGCCGAGTTGTAGTCGGCAGTCTATCTTGAGAACAATTGTTTTTACATGACAGATAGTGGGCTAAGAATAACTTTGCTCAAACCATTTGGTAAAACTGCTCAATGGACGAGCCGATTTTCACCCCGGCAATTTTGTTGAACCTCCCACTTCTCCGGTGGTCATTCTCAAGGAGTTGGTGGCTAAGTTGTACCGGGAACAAAATAAAGTGCAGGATCTGTTGGGGGCCATGGGCTACGCCCTGCGGAGTTTACATAATCTGAATCAGTTTCTGGAATTGACCCCTCTGATGGCCACCAGGGTCACCGATGCCGATGGCAGTGCTTTAGTTCTAATGAGAGAGGGGGAAATATCTATCTTTGAACAAATCCATGGCCACAAAAATAGTCTTAAGGGCACCATTAAAGGCGCTTTGCAGAAGGCCCGCCAGGTTAACCTCACATTGGATTCTTCCACTGTCCTGAGTTATTTTGACCGTCAATTGCGCCAAGAATTACCGGCGATCGCCTGTTATAACACCCCCATTTTGAGT |
| >Cm^R^ | CGCAGAAAGGCCCACCCGAAGGTGAGCCAGTGTGACTCTAGTAGAGAGCGTTCACCGACAAACAACAGATAAAACGAAAGGCCCAGTCTTTCGACTGAGCCTTTCGTTTTATTTGATGCCTGGTTACGCCCCGCCCTGCCACTCATCGCAGTACTGTTGTAATTCATTAAGCATTCTGCCGACATGGAAGCCATCACAGACGGCATGATGAACCTGAATCGCCAGCGGCATCAGCACCTTGTCGCCTTGCGTATAATATTTGCCCATGGTGAAAACGGGGGCGAAGAAGTTGTCCATATTGGCCACGTTTAAATCAAAACTGGTGAAACTCACCCAGGGATTGGCTGAGACGAAAAACATATTCTCAATAAACCCTTTAGGGAAATAGGCCAGGTTTTCACCGTAACACGCCACATCTTGCGAATATATGTGTAGAAACTGCCGGAAATCGTCGTGGTATTCACTCCAGAGCGATGAAAACGTTTCAGTTTGCTCATGGAAAACGGTGTAACAAGGGTGAACACTATCCCATATCACCAGCTCACCGTCTTTCATTGCCATACGGAATTCCGGATGAGCATTCATCAGGCGGGCAAGAATGTGAATAAAGGCCGGATAAAACTTGTGCTTATTTTTCTTTACGGTCTTTAAAAAGGCCGTAATATCCAGCTGAACGGTCTGGTTATAGGTACATTGAGCAACTGACTGAAATGCCTCAAAATGTTCTTTACGATGCCATTGGGATATATCAACGGTGGTATATCCAGTGATTTTTTTCTCCATTTTAGCTTCCTTAGCTCCTGAAAATCTCGATAACTCAAAAAATACGCCCGGTAGTGATCTTATTTCATTATGGTGAAAGTTGGAACCTCTTACGTGCCGATCA |
| >Spe^R^ | CGCAGAAAGGCCCACCCGAAGGTGAGCCAGTGTGACTCTAGTAGAGAGCGTTCACCGACAAACAACAGATAAAACGAAAGGCCCAGTCTTTCGACTGAGCCTTTCGTTTTATTTGATGCCTGGTTATTTGCCGACTACCTTGGTGATCTCGCCTTTCACGTAGTGGACAAATTCTTCCAACTGATCTGCGCGCGAGGCCAAGCGATCTTCTTCTTGTCCAAGATAAGCCTGTCTAGCTTCAAGTATGACGGGCTGATACTGGGCCGGCAGGCGCTCCATTGCCCAGTCGGCAGCGACATCCTTCGGCGCGATTTTGCCGGTTACTGCGCTGTACCAAATGCGGGACAACGTAAGCACTACATTTCGCTCATCGCCAGCCCAGTCGGGCGGCGAGTTCCATAGCGTTAAGGTTTCATTTAGCGCCTCAAATAGATCCTGTTCAGGAACCGGATCAAAGAGTTCCTCCGCCGCTGGACCTACCAAGGCAACGCTATGTTCTCTTGCTTTTGTCAGCAAGATAGCCAGATCAATGTCGATCGTGGCTGGCTCGAAGATACCTGCAAGAATGTCATTGCGCTGCCATTCTCCAAATTGCAGTTCGCGCTTAGCTGGATAACGCCACGGAATGATGTCGTCGTGCACAACAATGGTGACTTCTACAGCGCGGAGAATCTCGCTCTCTCCAGGGGAAGCCGAAGTTTCCAAAAGGTCGTTGATCAAAGCTCGCCGCGTTGTTTCATCAAGCCTTACGGTCACCGTAACCAGCAAATCAATATCACTGTGTGGCTTCAGGCCGCCATCCACTGCGGAGCCGTACAAATGTACGGCCAGCAACGTCGGTTCGAGATGGCGCTCGATGACGCCAACTACCTCTGATAGTTGAGTCGATACTTCGGCGATCACCGCTTCCCTCAT |
| >*hfq* | ATGGCTAAGGGGCAATCTTTACAAGATCCGTTCCTGAACGCACTGCGTCGGGAACGTGTTCCAGTTTCTATTTATTTGGTGAATGGTATTAAGCTGCAAGGGCAAATCGAGTCTTTTGATCAGTTCGTGATCCTGTTGAAAAACACGGTCAGCCAGATGGTTTACAAGCACGCGATTTCTACTGTTGTCCCGTCTCGCCCGGTTTCTCATCACAGTAACAACGCCGGTGGCGGTACCAGCAGTAACTACCATCATGGTAGCAGCGCGCAGAATACTTCCGCGCAACAGGACAGCGAAGAAACCGAATAA |
| >*micC* | TTTCTGTTGGGCCATTGCATTGCCACTGATTTTCCAACATATAAAAAGACAAGCCCGAACAGTCGTCCGGGCTTTTTTT |
| >T*rbcL* | ACCGGTGTTTGGATTGTCGGAGTTGTACTCGTCCGTTAAGGATGAACAGTTCTTCGGGGTTGAGTCTGCTAACTAATTAGCCATTAACAGCGGCTTAACTAACAGTTAGTCATTGGCAATTGTCAAAAAATTGTTAATCAGCCAAAACCCACTGCTTACTGATGTTCAACTTCGACAGC |
| >multiple cloning sites | ACTCGAGAAGATCTTGATATCCATATGAGGCCTAATCTAGATGCATTCGCGAGGGTACC |
| >PT1 | AGGAGGAATTAACCATGCAGTGGTGGTGGTGGTGGTGC |
| >PT2 | GCACCACCACCACCACCACTGCATGGTTAATTCCTCCT |
| >*lacZ* | ATGATTACGGATTCACTGGCCGTCGTTTTACAACGTCGTGACTGGGAAAACCCTGGCGTTACCCAACTTAATCGCCTTGCAGCACATCCCCCTTTCGCCAGCTGGCGTAATAGCGAAGAGGCCCGCACCGATCGCCCTTCCCAACAGTTGCGCAGCCTGAATGGCGAATGGCGCTTTGCCTGGTTTCCGGCACCAGAAGCGGTGCCGGAAAGCTGGCTGGAGTGCGATCTTCCTGAGGCCGATACTGTCGTCGTCCCCTCAAACTGGCAGATGCACGGTTACGATGCGCCCATCTACACCAACGTGACCTATCCCATTACGGTCAATCCGCCGTTTGTTCCCACGGAGAATCCGACGGGTTGTTACTCGCTCACATTTAATGTTGATGAAAGCTGGCTACAGGAAGGCCAGACGCGAATTATTTTTGATGGCGTTAACTCGGCGTTTCATCTGTGGTGCAACGGGCGCTGGGTCGGTTACGGCCAGGACAGTCGTTTGCCGTCTGAATTTGACCTGAGCGCATTTTTACGCGCCGGAGAAAACCGCCTCGCGGTGATGGTGCTGCGCTGGAGTGACGGCAGTTATCTGGAAGATCAGGATATGTGGCGGATGAGCGGCATTTTCCGTGACGTCTCGTTGCTGCATAAACCGACTACACAAATCAGCGATTTCCATGTTGCCACTCGCTTTAATGATGATTTCAGCCGCGCTGTACTGGAGGCTGAAGTTCAGATGTGCGGCGAGTTGCGTGACTACCTACGGGTAACAGTTTCTTTATGGCAGGGTGAAACGCAGGTCGCCAGCGGCACCGCGCCTTTCGGCGGTGAAATTATCGATGAGCGTGGTGGTTATGCCGATCGCGTCACACTACGTCTGAACGTCGAAAACCCGAAACTGTGGAGCGCCGAAATCCCGAATCTCTATCGTGCGGTGGTTGAACTGCACACCGCCGACGGCACGCTGATTGAAGCAGAAGCCTGCGATGTCGGTTTCCGCGAGGTGCGGATTGAAAATGGTCTGCTGCTGCTGAACGGCAAGCCGTTGCTGATTCGAGGCGTTAACCGTCACGAGCATCATCCTCTGCATGGTCAGGTCATGGATGAGCAGACGATGGTGCAGGATATCCTGCTGATGAAGCAGAACAACTTTAACGCCGTGCGCTGTTCGCATTATCCGAACCATCCGCTGTGGTACACGCTGTGCGACCGCTACGGCCTGTATGTGGTGGATGAAGCCAATATTGAAACCCACGGCATGGTGCCAATGAATCGTCTGACCGATGATCCGCGCTGGCTACCGGCGATGAGCGAACGCGTAACGCGAATGGTGCAGCGCGATCGTAATCACCCGAGTGTGATCATCTGGTCGCTGGGGAATGAATCAGGCCACGGCGCTAATCACGACGCGCTGTATCGCTGGATCAAATCTGTCGATCCTTCCCGCCCGGTGCAGTATGAAGGCGGCGGAGCCGACACCACGGCCACCGATATTATTTGCCCGATGTACGCGCGCGTGGATGAAGACCAGCCCTTCCCGGCTGTGCCGAAATGGTCCATCAAAAAATGGCTTTCGCTACCTGGAGAGACGCGCCCGCTGATCCTTTGCGAATACGCCCACGCGATGGGTAACAGTCTTGGCGGTTTCGCTAAATACTGGCAGGCGTTTCGTCAGTATCCCCGTTTACAGGGCGGCTTCGTCTGGGACTGGGTGGATCAGTCGCTGATTAAATATGATGAAAACGGCAACCCGTGGTCGGCTTACGGCGGTGATTTTGGCGATACGCCGAACGATCGCCAGTTCTGTATGAACGGTCTGGTCTTTGCCGACCGCACGCCGCATCCAGCGCTGACGGAAGCAAAACACCAGCAGCAGTTTTTCCAGTTCCGTTTATCCGGGCAAACCATCGAAGTGACCAGCGAATACCTGTTCCGTCATAGCGATAACGAGCTCCTGCACTGGATGGTGGCGCTGGATGGTAAGCCGCTGGCAAGCGGTGAAGTGCCTCTGGATGTCGCTCCACAAGGTAAACAGTTGATTGAACTGCCTGAACTACCGCAGCCGGAGAGCGCCGGGCAACTCTGGCTCACAGTACGCGTAGTGCAACCGAACGCGACCGCATGGTCAGAAGCCGGGCACATCAGCGCCTGGCAGCAGTGGCGTCTGGCGGAAAACCTCAGTGTGACGCTCCCCGCCGCGTCCCACGCCATCCCGCATCTGACCACCAGCGAAATGGATTTTTGCATCGAGCTGGGTAATAAGCGTTGGCAATTTAACCGCCAGTCAGGCTTTCTTTCACAGATGTGGATTGGCGATAAAAAACAACTGCTGACGCCGCTGCGCGATCAGTTCACCCGTGCACCGCTGGATAACGACATTGGCGTAAGTGAAGCGACCCGCATTGACCCTAACGCCTGGGTCGAACGCTGGAAGGCGGCGGGCCATTACCAGGCCGAAGCAGCGTTGTTGCAGTGCACGGCAGATACACTTGCTGATGCGGTGCTGATTACGACCGCTCACGCGTGGCAGCATCAGGGGAAAACCTTATTTATCAGCCGGAAAACCTACCGGATTGATGGTAGTGGTCAAATGGCGATTACCGTTGATGTTGAAGTGGCGAGCGATACACCGCATCCGGCGCGGATTGGCCTGAACTGCCAGCTGGCGCAGGTAGCAGAGCGGGTAAACTGGCTCGGATTAGGGCCGCAAGAAAACTATCCCGACCGCCTTACTGCCGCCTGTTTTGACCGCTGGGATCTGCCATTGTCAGACATGTATACCCCGTACGTCTTCCCGAGCGAAAACGGTCTGCGCTGCGGGACGCGCGAATTGAATTATGGCCCACACCAGTGGCGCGGCGACTTCCAGTTCAACATCAGCCGCTACAGTCAACAGCAACTGATGGAAACCAGCCATCGCCATCTGCTGCACGCGGAAGAAGGCACATGGCTGAATATCGACGGTTTCCACATGGGGATTGGTGGCGACGACTCCTGGAGCCCGTCAGTATCGGCGGAATTCCAGCTGAGCGCCGGTCGCTACCATTACCAGTTGGTTTGGTGTCAGAAGTAA |
| >P*cpc560* | ACCTGTAGAGAAGAGTCCCTGAATATCAAAATGGTGGGATAAAAAGCTCAAAAAGGAAAGTAGGCTGTGGTTCCCTAGGCAACAGTCTTCCCTACCCCACTGGAAACTAAAAAAACGAGAAAAGTTCGCACCGAACATCAATTGCATAATTTTAGCCCTAAAACATAAGCTGAACGAAACTGGTTGTCTTCCCTTCCCAATCCAGGACAATCTGAGAATCCCCTGCAACATTACTTAACAAAAAAGCAGGAATAAAATTAACAAGATGTAACAGACATAAGTCCCATCACCGTTGTATAAAGTTAACTGTGGGATTGCAAAAGCATTCAAGCCTAGGCGCTGAGCTGTTTGAGCATCCCGGTGGCCCTTGTCGCTGCCTCCGTGTTTCTCCCTGGATTTATTTAGGTAATATCTCTCATAAATCCCCGGGTAGTTAACGAAAGTTAATGGAGATCAGTAACAATAACTCTAGGGTCATTACTTTGGACTCCCTCAGTTTATCCGGGGGAATTGTGTTTAAGAAAATCCCAACTCATAAAGTCAAGTAGGAGATTAATTCA |
| >P*psbA2* | TATCAATAAGTATTAGGTATATGGATCATAATTGTATGCCCGACTATTGCTTAAACTGACTGACCACTGACCTTAAGAGTAATGGCGTGCAAGGCCCAGTGATCAATTTCATTATTTTTCATTATTTCATCTCCATTGTCCCTGAAAATCAGTTGTGTCGCCCCTCTACACAGCCCAGAACTATGGTAAAGGCGCACGAAAAACCGCCAGGTAAACTCTTCTCAACCCCCAAAACGCCCTCTGTTTACCCATGGAAAAAACGACAATTACAAGAAAGTAAAACTTATGTCATCTATAAGCTTCGTGTATATTAACTTCCTGTTACAAAGCTTTACAAAACTCTCATTAATCCTTTAGACTAAGTTTAGTCAGTTCCAATCTGAACATCGACAAATACATAAGGAATTATAACCAA |
| >P*psbA2M* | TATCAATAAGTATTAGGTATATGGATCATAATTGTATGCCCGACTATTGCTTAAACTGACTGACCACTGACCTTAAGAGTAATGGCGTGCAAGGCCCAGTGATCAATTTCATTATTTTTCATTATTTCATCTCCATTGTCCCTGAAAATCAGTTGTGTCGCCCCTCTACACAGCCCAGAACTATGGTAAAGGCGCACGAAAAACCGCCAGGTAAACTCTTCTCAACCCCCAAAACGCCCTCTGTTTACCCATGGAAAAAACGACAATTACAAGAAAGTAAAACTTATGTCATCTATAAGCTTCGTGTATATTAACTTCCTGTTACAAAGCTTTACAAAACTCTCATTAATCCTTTAGACTAAGTTTAGTCAGTTCCAATCTGAACATCGACAAATACAT |
| >P*trcM* | ATGAGCTGTTGACAATTAATCATCCGGCTCGTATAATGTGTGGAATTGTGAGCGGATAACAATTTCACACA |
| >P*trc*- theophylline riboswitch | ATGAGCTGTTGACAATTAATCATCCGGCTCGTATAATGTGTGGAATTGTGAGCGGATAACAATTTCATACGCTCACAATTGGTACCGGTGATACCAGCATCGTCTTGATGCCCTTGGCAGCACCCTGCTAAGGAGGCAACAAG |

**Table S3. Sequences used for calculating binding energies between sRNA and its target.**

| >*slr1511* | UUGAAUACCCUUGGCAGUGGCCUCAAAAUUAUUGGCAGUGGCACGGCGAUCGCCGACCAGAGUUUAACAAAUCAAGACCUGAGCAACAUUGUCGAGACCUCCGACGAGUGGAUCCAGUCCCGCACGGGGAUGCGCCAACGCUACAUUUGUUCGGCCCAGGAAAAUUUGGCCAGUUUGGGGGUAAAGGCCGGCCAAAAAGCCCUGGCCAUGGCGGGACUACAACCGGAAGACCUGGAUUUAAUUAUUCUGGCCACCUCCACCCCCGAUGAUUUAUUUGGCACUGCAGCCCAAAUUCAAGGGGGAUUGGGGGCCACCAGAGCCUUUGCCUUUGACAUAACGGCGGCCUGUUCUGGUUUUGUGGUGGGGUUGAACGUAGCAGCCCAAUUUUUACGCACCGGGGUUUACCAAAGGGUUUUAAUAGUGGGAGGGGAUGUACUUUCCCGUUGGGUGGACUGGUCGGACCGCACCACCUGUGUGUUAUUCGGUGAUGGAGCCGGAGCCGUGGUGCUACAACGCCAAGCCCAGGACAAUUUGCUCGCCUUUGAAAUGUACACCGAUGGCACUGGCAAUGGCUGUCUUAAUUUGUCCUAUCAAGCAAACCCUCAACCUUUAACAGCAGAAAAAACCGUUGCCCAGGGCACCUAUCAAGCCAUCACCAUGAACGGCCGGGAAGUGUACCGUUUUGCCGUGGCUAAGGUGCCCGAAAUCAUCGAAAAAGUACUGUUUAAAGCCCAGUUGACCACCUCCGACUUGGACUGGGUAAUUCUUCACCAAGCCAAUCAACGCAUUAUGGAUGCGGUGGGCGAUCGCCUGGGUAUUCCAUCCGAGAAAAUCAUCAGUAAUGUAGGGGAAUACGGCAACACUUCGGCAGCUUCCAUUCCCCUGGCUUUGGAUCAAGCGGUGCGGGAAGGGAAAAUUAAAGAGGGAGACCUAAUCGCCCUGGCCGGCUUUGGGGCCGGCUUAACCUGGGCUGCUAGCAUAGUGCGGUGGUAA |
| --- | --- |
| >*slr2023* | AUGAAAACUGCAUGGGUAUUCCCCGGCCAAGGUUCCCAAGCUGUGGGCAUGGGGGUAGAUCUGCUCUCAACGGCGAUCGCCAAGGAAAAAUAUCAGCAAGCCGAAGAAAUUCUUGGCUGGUCAGUGGUGGAAAAAUGCCAAGGGGAUGAAGCUAGCCUGGCCCUGACCCAAAAUACCCAACCCUGUUUGUACGUUAUUGAAGCAAUUUUGGCUGAUUUGCUCAGGGAUAAAGGUUUUCAGCCCGAUUACGUAGCCGGUCAUAGUCUGGGGGAAUAUUCCGCCCUCUACGCCGCUGGGGUAUUUGAUUUUGCCACGGGUUUGCAACUGGUUAAACAACGGUCAGAGGUGAUGGCCAGCGCUUCCGGGGGCAUGAUGGCCGCCCUGAUGAAGUUCGAUCAAACCCAGUUACAGCAAGCCCUGACGGAUAAUACAGAGGUUGUAUUAGCUAACGAUAACAGCCCCGAACAGGUAGUAAUUUCCGGUACUGUGGCCGGUGUAGAAGCAAUUUUAGCCAACGUCAAGGCACGCCGAGCCGUGCCUCUGAAGGUUUCCGGUGCUUUCCACUCCAGCUUUAUGGCCCAACCUUCCCAAUCCUUCGCCCAAACCCUGACAGCUUGCCAUUUCAACGACGCCACUGUGCCCGUACUCUCCAACGUUGAUCCUAGCCCUACCCAAAACGGCGAUCGCCUGAAAGAAAAACUAAUUCAACAAAUGACCGGUUCCGUGCGCUGGCGGGAAACCAUGGUUAACCUAGGAGAAAUUGGGGCAACGGAUUAUUGGGAAGUGGGCCCAGGCAAAGUACUGACUGGUCUAUGUAAACGAACUUGCCCUGAUCUGAACCUGAAAAAUAUUGGCCAAUUAGACGACUUGAAUUCCCUGUAG |
| >*antislr1511* | GAGGCCACUGCCAAGGGUAUUCAAUUUCUGUUGGGCCAUUGCAUUGCCACUGAUUUUCCAACAUAUAAAAAGACAAGCCCGAACAGUCGUCCGGGCUUUUUUU |
| >*antislr2023* | GGGGAAUACCCAUGCAGUUUUCAUUUUCUGUUGGGCCAUUGCAUUGCCACUGAUUUUCCAACAUAUAAAAAGACAAGCCCGAACAGUCGUCCGGGCUUUUUUU |
